# The Genetic Architecture of Barley Plant Stature

**DOI:** 10.3389/fgene.2016.00117

**Published:** 2016-06-24

**Authors:** Ahmad M. Alqudah, Ravi Koppolu, Gizaw M. Wolde, Andreas Graner, Thorsten Schnurbusch

**Affiliations:** ^1^HEISENBERG-Research Group Plant Architecture, Leibniz-Institute of Plant Genetics and Crop Plant ResearchGatersleben, Germany; ^2^Research Group Genome Diversity, Leibniz Institute of Plant Genetics and Crop Plant ResearchGatersleben, Germany

**Keywords:** barley, tillering, plant height, *vrs1*, *Ppd-H1*, GWAS

## Abstract

Plant stature in temperate cereals is predominantly controlled by tillering and plant height as complex agronomic traits, representing important determinants of grain yield. This study was designed to reveal the genetic basis of tillering at five developmental stages and plant height at harvest in 218 worldwide spring barley (*Hordeum vulgare* L.) accessions under greenhouse conditions. The accessions were structured based on row-type classes [two- vs. six-rowed] and photoperiod response [photoperiod-sensitive (*Ppd-H1*) vs. reduced photoperiod sensitivity (*ppd-H1*)]. Phenotypic analyses of both factors revealed profound between group effects on tiller development. To further verify the row-type effect on the studied traits, *Six-rowed spike 1* (*vrs1*) mutants and their two-rowed progenitors were examined for tiller number per plant and plant height. Here, wild-type (*Vrs1*) plants were significantly taller and had more tillers than mutants suggesting a negative pleiotropic effect of this row-type locus on both traits. Our genome-wide association scans further revealed highly significant associations, thereby establishing a link between the genetic control of row-type, heading time, tillering, and plant height. We further show that associations for tillering and plant height are co-localized with chromosomal segments harboring known plant stature-related phytohormone and sugar-related genes. This work demonstrates the feasibility of the GWAS approach for identifying putative candidate genes for improving plant architecture.

## Introduction

Tillering is one of the key components for improving grain yield in temperate cereals, such as wheat (*Triticum aestivum* L.) and barley (Sreenivasulu and Schnurbusch, 2012; Kebrom et al., 2013; Hussien et al., 2014). Cereals are able to maximize grain yield through increased tillering (Evers and Vos, 2013) and increasing the number of fertile tillers (bearing fertile spikes) was proposed as one of the most important components for grain yield in wheat and barley (Sreenivasulu and Schnurbusch, 2012; Xie et al., 2016). Variation in tillering was attributed to genetic variation between barley genotypes in tiller production (Abeledo et al., 2004; Alqudah and Schnurbusch, 2014), or genetic variation in pre-anthesis phase duration in a bi-parental population (Borras et al., 2009), or partially to the environmental influence (Abeledo et al., 2004; Borras et al., 2009). Previous studies on tillering mainly focused on the final tiller number at harvest but a few studies focused on tillering at different developmental stages, such as Borras et al. (2009); however, until now no study has documented the natural variation of tillering at pre-anthesis stages.

The generation of additional side-tillers (i.e., tillering) requires tiller bud formation and outgrowth, which is a complex developmental process under the control of genetic factors, environment, and phytohormone action (Kebrom et al., 2013). In barley, bud outgrowth into tillers (side-branches) happens sequentially after the three leaf stage (Kirby and Appleyard, 1987). The number of developing tiller buds or bud outgrowth is influenced by growing conditions such as light and water (Doust, 2007; Evers and Vos, 2013; Kebrom et al., 2013). Moreover, bud outgrowth in monocots and dicots is regulated by a complex and conserved pathway of phytohormones and their interactions including auxin, strigolactones [(SL; as suppressors)], cytokinins (as promoters by reducing auxin; Kebrom et al., 2013) and other hormones like brassinosteroids, abscisic acid (ABA), ethylene, and gibberellins (GAs). The role of phytohormones in bud outgrowth is well-reviewed (Evers and Vos, 2013; Kebrom et al., 2013) while Evers and Vos (2013) describe a mathematical model for tillering in cereals. So far, most of the phytohormonal knowledge in barley and wheat tillering regulation are based on results extrapolated from other grass species, such as rice and maize; however, hormonal pathways regulating bud outgrowth are not yet fully understood.

Recently, several studies highlighted the importance of sugars as a key component of plant stature regulations, for instance Evers (2015). The regulatory role of sugars during branching might be through regulating physiological mechanisms involving hormonal genes (Barbier et al., 2015). In any case, the role of sugars in shoot branching is not well-understood, and therefore, further genetic investigations on the role of sugars in shoot branching are required to reveal the underlying regulatory network of shoot branching in cereals.

Understanding the mechanisms of tillering may help to better understand and modify crop architecture in order to achieve better yield (Sreenivasulu and Schnurbusch, 2012). Several genes regulating tiller formation have already been identified and characterized such as *TEOSINTE BRANCHED 1* (*TB1*) in maize, which is under the control of SL, inhibit bud outgrowth by regulating maize *GRASSY TILLERS 1* (*GT1*; Kebrom et al., 2013). *INTERMEDIUM-SPIKE C* (*Int-C*; Ramsay et al., 2011) in barley, which is an ortholog of *TB1*; whereas barley *SIX-ROWED SPIKE 1* (*Vrs1*) is a homolog of *GT1* (Whipple et al., 2011). Even though, these genes inhibit lateral growth (branching), they do so in a different developmental context (Kebrom et al., 2013).

Decreasing barley plant height was the main strategy for improving grain yield and harvest index through reduced lodging (Bezant et al., 1996), using *SEMI-DWARF 1* (*sdw1* or *denso*) in Europe (EU) and East Asia (EA; Hellewell et al., 2000). Wang et al. (2010) found that dwarfing genes in barley have negative impact on spike agronomical traits such as spike length and grain density. The relationship between plant height and heading date was documented by Lin et al. (1995), where three alleles at the *sdw1* locus were associated with delay in heading (Hellewell et al., 2000) and some other alleles are day-length sensitive (Wang et al., 2010). Recently, Wang et al. (2014) found a new plant height QTL that positively affects barley agronomic traits and grain yield. Through Genome-Wide Association Studies (GWAS), Pasam et al. (2012) and Pauli et al. (2014) detected many QTL for barley plant height overlapping with previously mapped QTL and known genes. However, natural variation in plant height is still insufficient to understand the importance of this trait with respect to other agronomical traits. Thus, tools like GWAS analyses using high density genetic maps based upon different population structures are key to increase our knowledge concerning genetic factors controlling plant height.

So far several barley tillering mutant loci were identified, including *uniculme4* (*cul4*, Tavakol et al., 2015), *many noded dwarf6/densinodosum6* (*mnd6/den6*, Dabbert et al., 2010), *uniculme2* (*cul2*), *intermedium spike-m* (*int-m*) *intermedium spike-b* (*int-b*; Babb and Muehlbauer, 2003), *granum-a* (*gra-a;* Dabbert et al., 2010), and *absent lower laterals* (*als;* Dabbert et al., 2009), which also affect other barley plant architectural traits. In addition to *sdw1*, other plant height mutants are available, including *sdw2-4* and *short culm 1*(*hcm*; Borner et al., 1999; Franckowiak et al., 2005). Functional interaction studies of these mutants showed pleiotropic or epistatic effects between plant height and tiller development such as *gra-a* (Dabbert et al., 2010). Therefore, studying these traits in diverse barley collection can potentially explain the interconnection between these traits.

In barley, *Vrs1* is the major gene controlling the row-type of the spike (Komatsuda et al., 2007). In its functional form, *Vrs1* produces the two-rowed spike phenotype; while mutations in *Vrs1* result in the six-rowed spike phenotype. In our previous study we found substantial differences between two- and six-row barleys in terms of tiller number under various growth conditions with high heritability values (Alqudah and Schnurbusch, 2014). Very recently, Liller et al. (2015) similarly found that the allelic status at *vrs1* pleiotropically affected tiller number. Furthermore, *PHOTOPERIOD RESPONSE LOCUS 1* (*Ppd-H1*) is the key regulator of heading time in barley (Turner et al., 2005). Karsai et al. (1999) studied the effect of *Ppd-H1* on agronomical traits including tillering and plant height in a bi-parental barley mapping population. To the best of our knowledge, no research was performed to identify the natural variation of tillering and plant height based on row-type classes and allelic status at *Ppd-H1* in barley. Thus, this study was designed to detect QTL underlying natural variation of tiller number per plant at different pre-anthesis stages and plant height at harvest based upon differences in row-type and photoperiod response by phenotyping a worldwide spring barley collection under controlled greenhouse (GH) conditions. The GWAS analysis using a 9k gene-based single nucleotide polymorphisms (SNPs) chip (Comadran et al., 2012) provided an unprecedented genetic resolution for the studied traits. The strategy of phenotyping the plants at pre-anthesis stages emphasized that present genetic variation of tillering could be genetically dissected. In this study, development stage-specific QTL i.e., QTL that have not been reported before were detected for tillering and plant height. Apart from this, several putative orthologous barley genes (characterized for tillering and plant height in other species) were genetically mapped onto barley chromosomes based on SNP marker associations obtained from our GWAS study.

## Materials and methods

### The collection and population structure

A collection of 218 spring barley worldwide accessions was used in this study that includes 125 two- and 93 six-rowed accessions (Pasam et al., 2012 and Table S1). Moreover, the collection was divided into two groups based on allelic variation at the *Ppd-H1* locus (SNP22, G/T, Turner et al. (2005) and Sharma et al., in preparation), 95 photoperiod-sensitive (*Ppd-H1*) and 123 accessions carrying the reduced photoperiod sensitivity (*ppd-H1*) allele (Alqudah et al., 2014). The collection was structured using 6355 polymorphic SNPs. The collection includes 149 cultivars, 57 landraces and 18 breeding lines previously described by Haseneyer et al. (2010).

### Genotyping

Genotyping of this collection was performed using a genome-wide high-density 9K SNPs chip from Illumina^*TM*^ that assayed 7842 SNPs (Comadran et al., 2012). The markers that passed minor allele frequency (MAF) ≥0.05 were used in association analysis (6355 SNPs, Table S2). Finally, we used 4323, 4320, 4228, and 4050 SNPs for GWAS analysis of two-rowed, six-rowed, *Ppd-H1*, and *ppd-H1*groups, respectively. On average about 4200 SNPs per accession were scored and around 210 accessions per marker were used in analysis. We used genetic marker positions anchored by physical map positions SNPs markers generated based on Barke × Morex RILs POPSEQ population (Mascher et al., 2013).

### Phenotypic data

Seeds from each of the 218 spring barley accessions were grown for 10 days under controlled GH condition (LD condition, 16/8 h day/night and ~20/16°C day/night). Thirty seedlings of each accession were grown in 0.5-L pots (one plant per pot; 9-cm pot diameter and 9-cm height) in the GH. Previous tests by Alqudah and Schnurbusch (2014) showed that this pot size effectively restricted excessive tillering and enabled to genetically evaluate single plant potential for tillering under GH conditions. Pots were randomized three times per week to reduce border and temperature-gradient effects on plant growth and development. The phenotypic experiments were performed from September 2011 to April 2012 in eight consecutive batches due to limited GH space and feasibility of workload. The experiment had a completely randomized design with 30 plants per accession. Details about growth conditions, experimental setup and phenotyping for dissecting the pre-anthesis phase, i.e., developmental stages [awn primordium (AP, Z31–33); Tipping (TIP, Z49); heading (HD, Z55); anther extrusion (AE, Z65)]; can be found in Alqudah and Schnurbusch (2014) and Alqudah et al. (2014). The total tiller number per plant was recorded from three plants/accession (each plant was considered as a biological replicate) at each developmental stages (AP, TIP, HD, and AE), while at harvest (Hrv) total tillers from six biological replicates (plants) were grouped as productive (tiller, carrying spike) and non-productive tillers (tiller, without spike). Plant height data were collected from six biological replicates (plants) at Hrv as the distance between soil and the top of the plant without spike. The *vrs1* mutants of Barke, Bonus, and Foma were used in this study to collect tillering data at two developmental stages (Z37, flag leaf just visible and heading time, Z55). Barke mutant (8408-1) was described by Gottwald et al. (2009); whereas *vrs1* mutants from Bonus (*hex-v.03*) and Foma (*Int-d.12*) were described by Komatsuda et al. (2007). Phenotypic data of 218 accessions were analyzed by REML (Residual Maximum Likelihood) and BLUEs (Best Linear Unbiased Estimates) to estimate each accession's phenotypic mean, which in turn were used in the association analysis (SAS, 2006). Fisher's least significant difference (LSD) was used to compare between groups (i.e., two- vs. six-rowed and photoperiod-sensitive vs. reduced photoperiod sensitivity) and to compare between genotypes with mutants at the probability level *P* ≤ 0.05. Broad-sense heritability for traits in each group was calculated across growing times as the ratio between the genetic variance and the phenotypic variance which includes genotypic by growing times (environment) interaction variance and error variance components using PROC VARCOMP (SAS, 2006).

### Genome-wide association study (GWAS) analysis

GWAS of groups was performed using their corresponding genotype and phenotype datasets. A mixed linear model (MLM) using GenStat 16 (Genstat, 2014) was used to calculate associations between estimated phenotypic traits (BLUEs) and each single marker. Association analysis in MLM was performed using single trait association analysis with Eigen-analysis as correction of population structure and controlling false positive associations (Genstat, 2014). For detecting significant associations, we considered a threshold *P-value* of 0.01 (i.e., –log_10_ *P* ≥ 2) in all traits. A multiple test, i.e., the false discovery rate (FDR), was calculated using GenStat 16 (Genstat, 2014) to determine the significance level of the SNP *P-value* at < 0.05 to exclude false-positive associations (Storey and Tibshirani, 2003). Through this conservative method, we tightly set the significance level of the SNP *P-value* providing highly significant associations (−log_10_ *P* ≥ FDR). FDR approach is strictly used to validate the associations in complex traits such as heading date (Alqudah et al., 2014; Pauli et al., 2014). Allele effects were estimated relative to the performance of cultivar “Mansholt zweizeilig” for six-rowed and *Ppd-H1* groups and cultivar “Isaria” for two-rowed and *ppd-H1* groups. We used SNP markers that passed the FDR threshold to determine highly associated QTL within confidence interval ±5 cM. The interval ±5 cM was found as an average linkage disequilibrium in this population (Pasam et al., 2012), so we used it as a confidence interval to determine highly associated QTL. Known tillering and plant height genes (bold and italicized) were genetically anchored and located according to the Barke × Morex RILs (POPSEQ) sequence contigs using IPK barley BLAST server, Gatersleben (http://webblast.ipk-gatersleben.de/barley/). More information about these genes, their genbank accession numbers, barley high confidence probability gene, and their genetic positions are shown in Table S3.

## Results

### The structure of a worldwide spring barley collection

Using 6355 polymorphic SNPs markers from 9k array, the collection of 218 worldwide spring barley accessions was separable into two subpopulations: (i) based on row-type classes (two- and six-rowed phenotypes; Figure S1A), and (ii) based on alleles for photoperiod response to long day conditions [photoperiod-sensitive, *Ppd-H1*, and one specific reduced photoperiod sensitivity allele, *ppd-H1*; SNP22, G/T, Turner et al. (2005) Figure S1B].

### Phenotypic variation of tillering at different developmental stages

**Figure 1:**
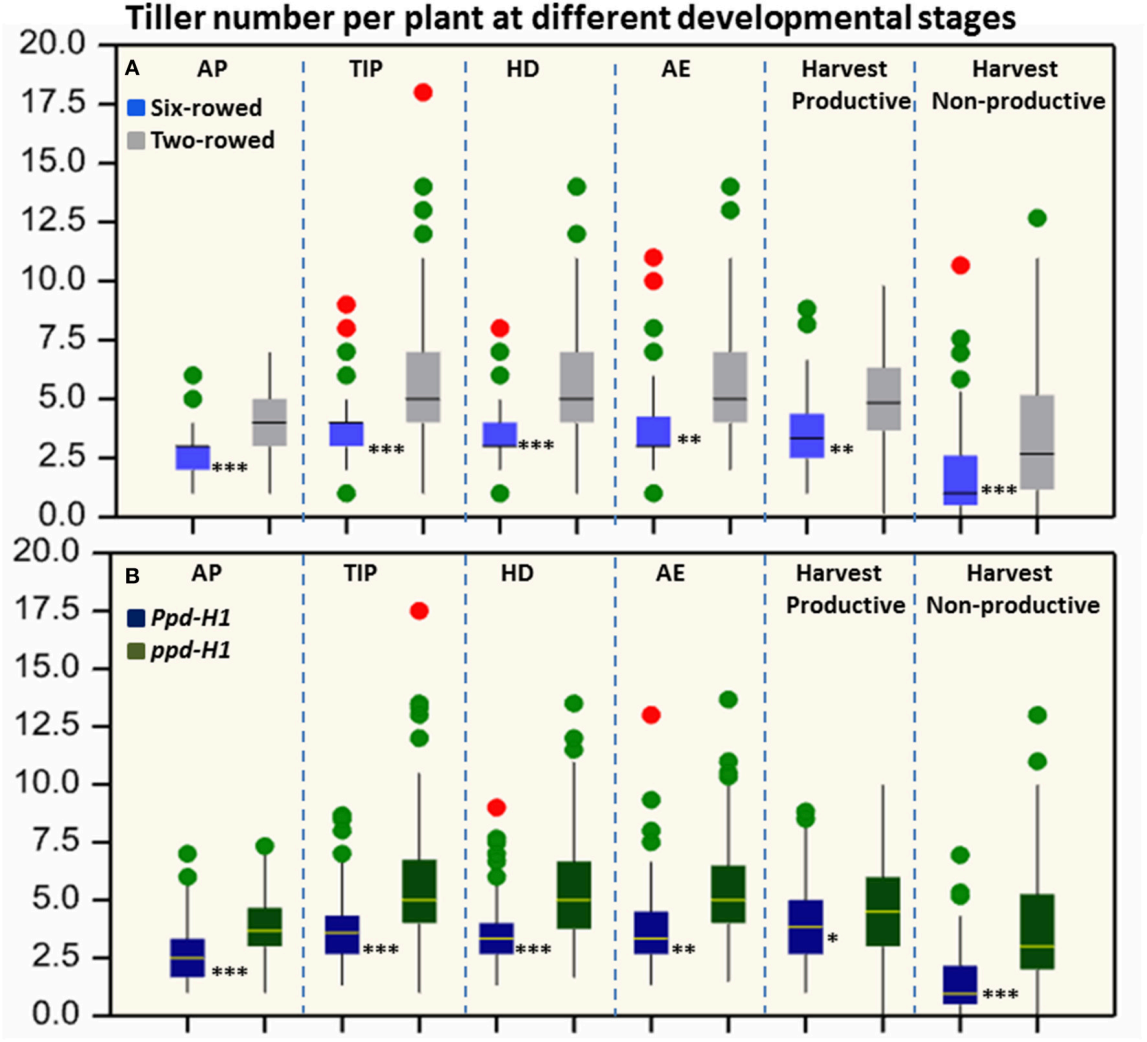
**Boxplots of total tiller number per plant in both row-type classes (A) and photoperiod groups (B)**. The degree of significance indicated as ^*^P, 0.05; ^**^P, 0.01; ^***^P, 0.001. Significant differences (*p* ≤ 0.05) were determined with a one-way ANOVA using LSD. Significant differences between the groups were calculated for each developmental stage separately. Three biological replicates were used from each accession at each pre-anthesis developmental stage and six biological replicates were used for counting productive and non-productive tiller at harvest stage. (*n* = 125 and 93 for two- and six-rowed barleys, respectively; and *n* = 95 and 123 for photoperiod sensitive and reduced photoperiod sensitivity barley, respectively). AP, awn primordium, Alqudah and Schnurbusch (2014); TIP, tipping, Z49; HD, heading, Z55; AE, anther extrusion, Z65; Hrv, Harvest, Zadoks et al. (1974). Developmental stages calculated based on thermal time °C × D^−1^ (GDD).

Significant differences (*P* ≤ 0.05) in tiller number per plant were found between row-type classes and photoperiod response groups. Two-rowed barley had significantly higher total number of tillers per plant compared to six-rowed at all developmental stages (Figure 1A). To further investigate the row-type effect on tillering, analyses of *vrs1* mutants and their progenitors were performed which showed that the total number of tillers per plant was significantly higher in two-rowed progenitors (Table 1). For photoperiod response groups, we found that plants with reduced photoperiod sensitivity (*ppd-H1*) had significantly more total tillers per plant at all developmental stages compared to photoperiod sensitive plants (*Ppd-H1*; Figure 1B). The variation within two-rowed and *ppd-H1* was larger than in other groups (Figures 1A,B). At harvest stage, the number of productive and non-productive tillers were significantly higher in two-rowed and *ppd-H1* groups (Figures 1A,B). Based on the origin of accessions in each group, EU accessions had more tillers per plant at pre-anthesis stages and non-productive tillers at Hrv in case of two-rowed, six-rowed and *Ppd-H1* groups; whereas the difference was not evident in the *ppd-H-1* group (Figures S2A–D). In terms of biological status, we found that improved cultivars had significantly higher number of total tillers per plant at pre-anthesis stages and non-productive tillers at Hrv (at *P* ≤ 0.05) than breeder's lines and landraces likely because of selection (Figure S3).

**Table 1:** **Total tiller number per plant and plant height in Barke, Bonus, and Foma (***Vrs1***) and their induced mutants Barke mutant (8408-1), ***hex-v.3***, and ***int-d.12*** (***vrs1***), respectively, at two developmental stages**.

**Genotype**	**Tiller per plant**		**Plant height (cm)**	
	**Z37^†^**	**Z55**	**Z37**	**Z55**
Barke	12.0a^*^	17.3a	46.3a	75.6a
8408-1 mutant	6.3b	11.0b	49.6a	67.3b
Bonus	14.3a	18.6a	43.3a	84.6a
*hex-v.3*	7.3b	11.3b	41.3a	71.3b
Foma	12.3a	20.3a	36.6b	55.3a
*int-d.12*	4.3b	12.0b	50.0a	59.6a
Wild type *Vrs1*	12.8a	18.7a	42.1a	71.7a
Mutant *vrs1*	5.9b	11.4b	46.9a	66.1b

† *Z37, flag leaf just visible; and Z55, heading time. Three biological replicates were used from each genotype at each developmental stage*.

* *different letters in each pair indicate there is significant difference at P = 0.05 according to the LSD test*.

### Phenotypic variation of plant height

**Figure 2:**
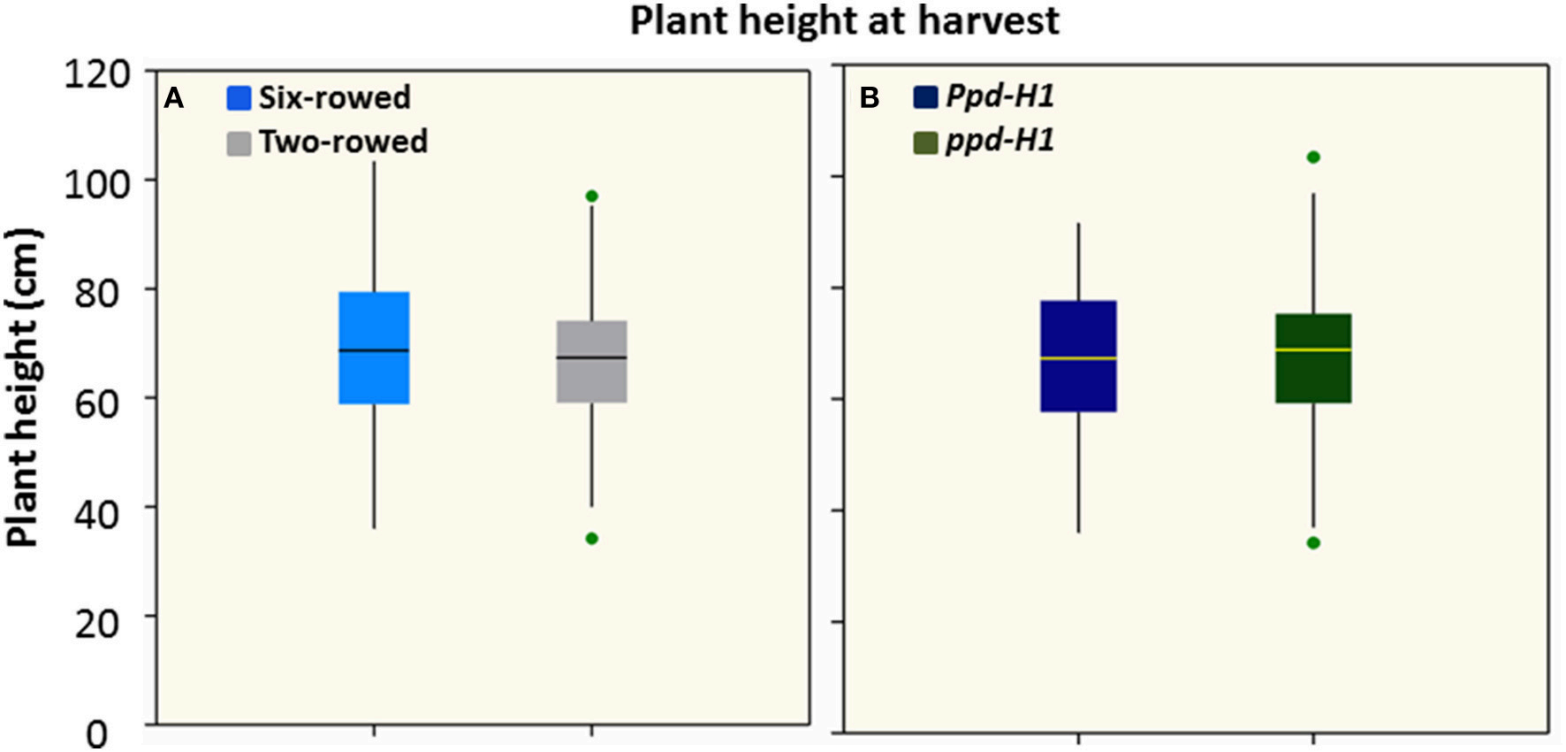
**Boxplots of plant height (cm) in both row-type classes (A) and photoperiod response groups (B)**. Significant differences (*p* ≤ 0.05) were determined with a one-way ANOVA using LSD. Significant differences between the groups were calculated harvest stage. Six biological replicates were used from each accession at harvest stage. (*n* = 125 and 93 for two- and six-rowed barleys, respectively; and *n* = 95 and 123 for photoperiod sensitive and reduced photoperiod sensitive barley, respectively).

Analysis of plant height at harvest did not show any significant difference between row-type classes and between photoperiod groups (*P* ≤ 0.05; Figures 2A,B). However, analyses of *vrs1* mutants and their progenitors found that wild-type plants were taller than mutants at HD stage (Z 55, Table 1). The geographical origins of the accessions showed significant differences (at *P* ≤ 0.05) in plant height within photoperiod response groups (Figure S4). AM accessions were the tallest in the *Ppd-H1* group; whereas these accessions were also shortest in the *ppd-H1* group (Figures S4C,D). In our study, we did not find any effect of biological status (at *P* ≤ 0.05) on plant height (Figure S5).

The phenotypic data of studied traits at different developmental stages for 218 accessions are available in Table S4. Interestingly, broad-sense heritability values for the traits studied (tiller number and plant height) ranged from high to very high in all groups (Table 2), indicating that they are predominantly genetically controlled.

**Table 2:** **Estimation of broad-sense heritability (***H***^**2**^) for tiller number per plant at different developmental stages and plant height at harvest**.

**Group**	**Tiller per plant**						**Plant height**
	**AP^†^**	**TIP**	**HD**	**AE**	**Hrv**		**Hrv**
					**Productive**	**Non-productive**	
Two-rowed	0.80	0.92	0.94	0.96	0.92	0.88	0.92
Six-rowed	0.70	0.94	0.90	0.85	0.92	0.90	0.95
*Ppd-H1*	0.78	0.90	0.93	0.87	0.90	0.85	0.93
*ppd-H1*	0.76	0.91	0.92	0.91	0.91	0.87	0.92

† *AP, awn primordium, Alqudah and Schnurbusch (2014); TIP, tipping, Z49; HD, heading, Z55; AE, anther extrusion, Z65; Hrv, Harvest, Zadoks et al. (1974). H^2^: broad-sense heritability for each group overall growing times based on accessions mean. n = 125 and 93 for two- and six-rowed barleys, respectively. n = 95 and 123 for barleys with photoperiod-sensitive and reduced photoperiod sensitivity, respectively. Developmental stages calculated based on thermal time °C × D^-1^ (GDD)*.

### Correlation analysis between thermal time of developmental stages and studied traits

**Figure 3:**
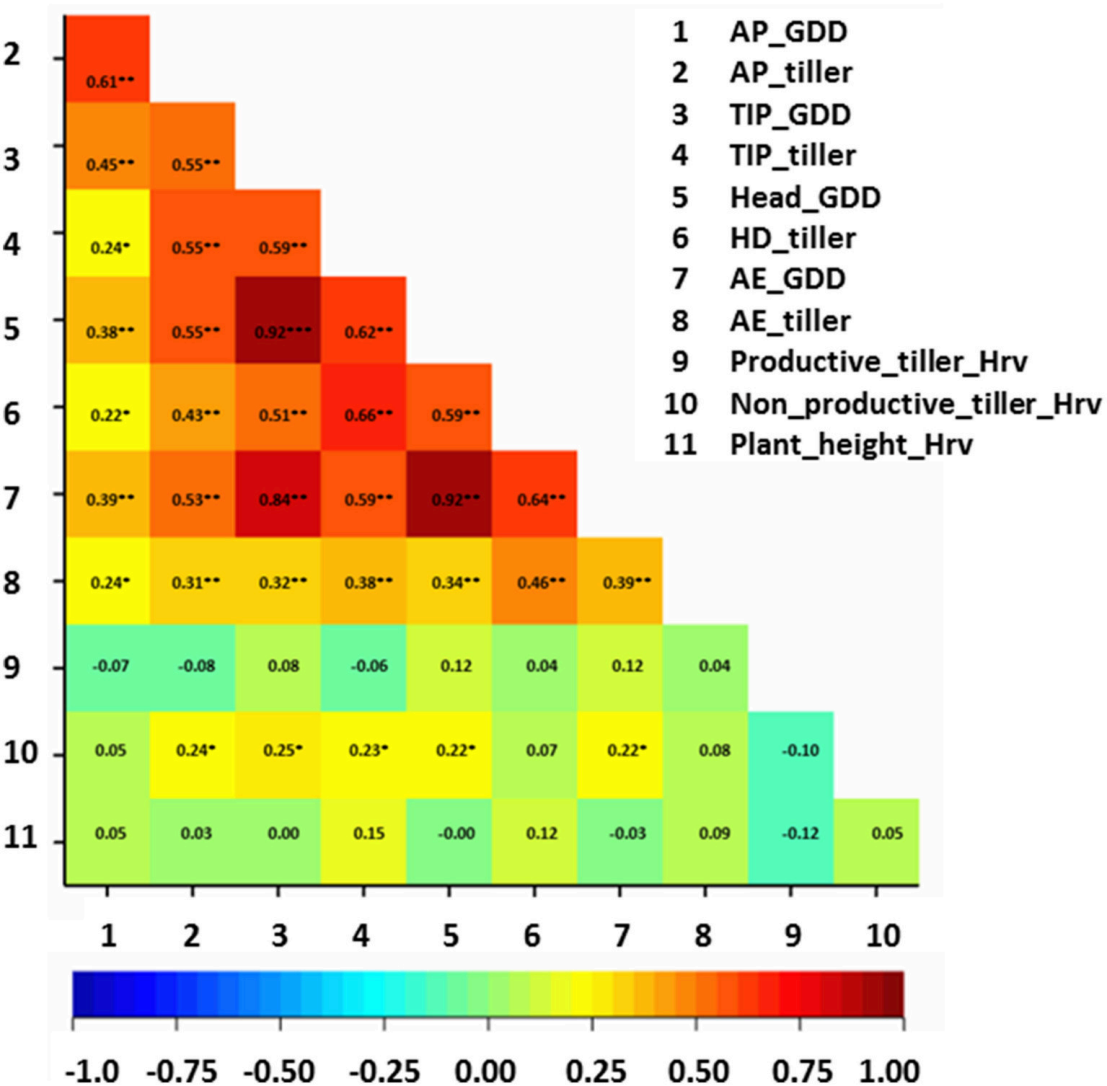
**Correlation matrix for the studied traits with growing-degree days (GGD)**. The degree of significance indicated as ^*^P, 0.05; ^**^P, 0.01; ^***^P, 0.001. AP, awn primordium, Alqudah and Schnurbusch (2014); TIP, tipping, Z49; HD, heading, Z55; AE, anther extrusion, Z65; Hrv, Harvest, Zadoks et al. (1974). Developmental stages calculated based on thermal time °C × D^−1^ (GDD).

Correlation analysis between studied traits and thermal time at developmental stages was performed on the whole collection (Figure 3). Generally, correlation values were moderate (*r* ≈ 0.6^**^) between total tiller number per plant and growing-degree days (GDD) at AP, TIP, and HD stages (Figure 3), while only low (*r* ≈ 0.4^**^) at AE. The correlation values between total number of tillers at pre-anthesis developmental stages (e.g., at AP and at TIP) and GDD ranged between 0.30^**^–0.65^**^ (Figure 3). There was no clear trend of correlations between productive tiller number at Hrv and GDD of pre-anthesis stages and total tiller number at these stages. In contrast, only weak correlations were obtained between non-productive tillers at Hrv and GDD of pre-anthesis stages and total tiller number at these stages (*r* ≈ 0.2^*^; Figure 3). For plant height, there were no correlations between GDD and plant height at different developmental stages (Figure 3). These findings suggest that longer phase duration may lead to more tillers during pre-anthesis phases and more non-productive tillering at Hrv.

### Natural variation of tillering

The major loci for row-type (*Vrs1*) and heading time (*Ppd-H1*) appear to be the key genetic determinants affecting tiller number in the whole collection (Figure S6A). These genes were consistently detectable during early pre-anthesis stages (AP, TIP, and HD). Therefore, GWAS was conducted for the four groups separately (two-rowed, six-rowed, *Ppd-H1, ppd-H1*).

### QTL detection for tillering within row-type groups

**Figure 4:**
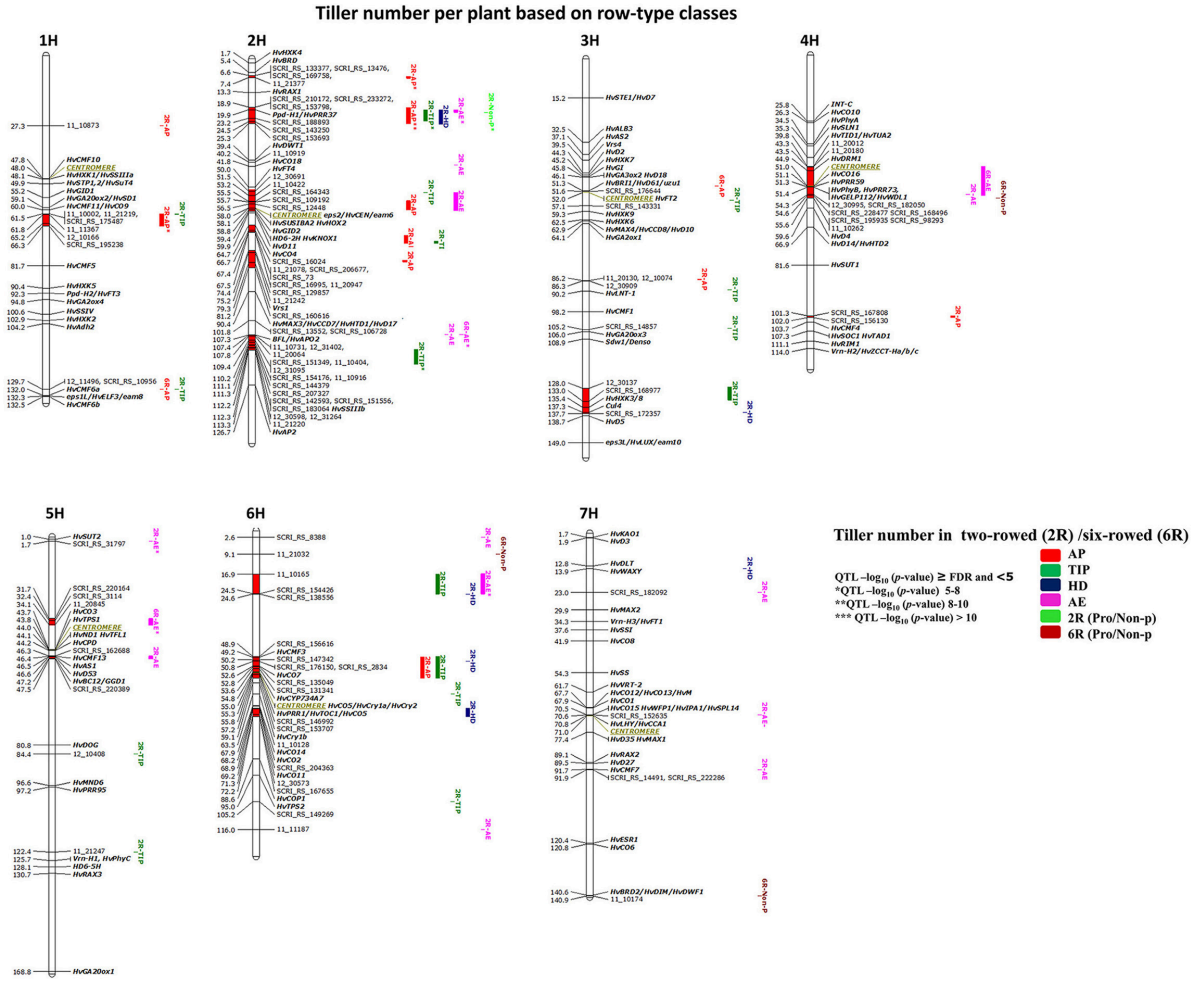
**Genetically anchored position of highly associated QTL for tiller number per plant at all barley developmental stages based on spike row-type (two-rowed/six-rowed) groups using 9K SNP markers**. Bold and italicized gene names indicate genetically anchored positions of known heading time and plant stature genes in the Barke x Morex RILs. Associated chromosomal regions are highlighted with different colors according to developmental stages. Red chromosomal areas indicate the range of significantly associated QTL (within confidence interval ±5 cM) which are exceeding FDR level of each developmental stage.

GWAS analysis for 125 two- and 93 six-rowed accessions was performed to study the natural variation within each group. We detected in total 53 significant marker-trait associations (≥FDR; Table 3) distributed across 15 chromosomal QTL regions (chromosomal region in red color). Only one six-rowed-specific QTL (5H 31.7–34.3 cM) was identified, while 14 QTL were two-rowed-specific (Figure 4). Plenty of natural genetic variation was found at pre-anthesis stages (AP, TIP, and HD; Figure 4).

**Table 3:** **False Discovery Rate (FDR) threshold (***P*** = 0.05) for tiller number per plant at each developmental stage and plant height at harvest in group of barley accessions**.

**Group**	**Tiller number per plant**						**Plant height**
	**AP^†^**	**TIP**	**HD**	**AE**	**Hrv**		**Hrv**
					**Productive**	**Non-productive**	
Two-rowed	3.44 (52)	3.46 (58)	3.44 (15)	3.52 (24)	3.70 (0)	4.28 (7)	3.49 (5)
Six-rowed	3.45 (1)	3.32 (0)	3.21 (0)	3.48 (8)	3.72 (0)	4.30 (3)	3.42 (17)
*Ppd-H1*	5.12 (2)	4.98 (4)	4.61 (3)	5.05 (2)	3.60 (72)	2.97 (0)	3.60 (1)
*ppd-H1*	4.51 (2)	4.75 (6)	3.71 (0)	5.21 (1)	4.06 (8)	3.10 (38)	3.41 (17)
Whole population							3.32 (6)

*SNPs exceeding FDR threshold are considered as highly significant SNPs*.

*Number of SNPs exceeding FDR threshold are shown in brackets*.

† *AP, awn primordium, (Alqudah and Schnurbusch, 2014; Alqudah et al., 2014); TIP, tipping, Z49; HD, heading, Z55; AE, anther extrusion, Z65; Hrv, Harvest, (Zadoks et al., 1974; Alqudah et al., 2014)*.

Through detailed association analysis, we found several interesting regions for tillering based on row-type classes. Six chromosomal regions have tillering effects, such as on 1H, 61.5–66.3 cM; 2H, 6.6–7.4 cM; 3HL, 128–137.7 cM; 4H, 101–102 cM; 5H, 31.7–34.1; and 6H, 16.9–24.6 cM (Figure 4) of which five regions are putatively novel QTL lacking known candidate genes. The chromosomal regions at 1H, 2H, and 4H strongly appeared at earlier stages (AP^*^ and TIP). We were unable to co-locate known genes for other significant chromosomal regions on 5H (31.7–34.1 cM), where we hypothesize that the *EARLY MATURITY* 7 (*eam7*) locus could underlie the 6H 17 cM QTL (Alqudah et al., 2014).

The highest significant marker effects were found for SNPs on 2H (19.9 cM), which co-localized with *Ppd-H1* at all pre-anthesis stages in two-rowed barleys (Figure 4), whereby the *Ppd-H1* group reduced the number of tillers per plant at AP, TIP and HD stages by −0.86, −1.32, and −0.68 tillers per plant, respectively. Another significant region is co-localized with the position of *Barley FLORICAULA/LEAFY* (*BFL*, 2HL 107.3 cM) and *SOLUBLE STARCH SYNTHASE* (*HvSSIIIb*, 2HL 112.1 cM, Figure 4,), which appeared at TIP, reducing tillering by ~−1.0 tiller per plant. Moreover, several chromosomal regions were precisely co-localized with genes in the centromeric region of 2H (~58 cM) with significant effects at AP and TIP [(e.g., 2H 58 cM, *CENTRORADIALIS* (*eps2/HvCEN/eam6)*, and *SUGAR SIGNALLING IN BARLEY 2 (HvSUSIBA2)*; 2H 59.4 cM, *HEADING DATE6* (*HD6-2H*); 2H 64.73 cM, *CONSTANS4* (*HvCO4*)]. The centromeric region on 2H also includes very interesting tillering-related genes like *GIBBERELLIN-INSENSITIVE DWARF2* (*HvGID2*); *KNOTTED1-LIKE HOMEOBOX1* (*HvKNOX1*); and *DWARF11* (*HvD11*); (Figure 4 and Table S3). However, the possibility of linkage is high in centromeric region, so we cannot be sure which gene(s) cause the phenotypic effect. Besides these associations, we found other associations close to putative heading time genes on 6H [(49.22 cM, *CCT MOTIF FAMILY*3 (*HvCMF3*); 52.62 cM, *HvCO7*; 54.2 cM, *CYTOCHROME P450 (HvCYP734A7)*; 55.38 cM, *ARABIDOPSIS PSEUDO-RESPONSE REGULATOR1/ TIMING OF CAB EXPRESSION1* (*HvPRR1/HvTOC1*); 59.06 cM, *CRYPTOCHROMES1b* (*HvCry1b*); 67.91 cM, *HvCO14*; 68.20 cM, *HvCO2;* and 69.2*, HvCO11*, Figure 4 and Table S3)].

Other interesting associations were also found around the centromeric region of 4H (51 cM) including *DORMANCY-ASSOCIATED1* (*HvDRM1*, 44.90 cM); *HvCO16, HvPRR59, HvPhyB/HvPRR73* (51.1-51.4 cM); *GDSL ESTERASE/LIPASE PROTEIN112, WILTED DWARF AND LETHAL1* (*HvGELP112/HvWDL1*, 51.4 cM; Figure 4), or on 5H (46.3–47.5 cM), which includes *HvCMF13, ASPARAGINE SYNTHASE1* (*HvAS1*), *HvD53*, and *BRITTLE CULM12/GIBBERELLIN-DEFICIENT DWARF1* (*HvBC12/GGD1*).

In this study we detected two interesting regions for non-productive tillering in six-rowed barleys on 7H (140.9 cM), which is close to *BRASSINOSTEROID DEFICIENT DWARF2/ DIMINUTO, DWARF1 (HvBRD2/HvDIM/HvDWF1*, 140.6 cM), and on 6H (9.1 cM). Findings in this section confirmed that there is plenty of variation in tillering especially at early developmental stages. Several associations are co-located with regions being associated with putative candidate genes while few appear to be novel.

### QTL detection for tillering within photoperiod response groups

**Figure 5:**
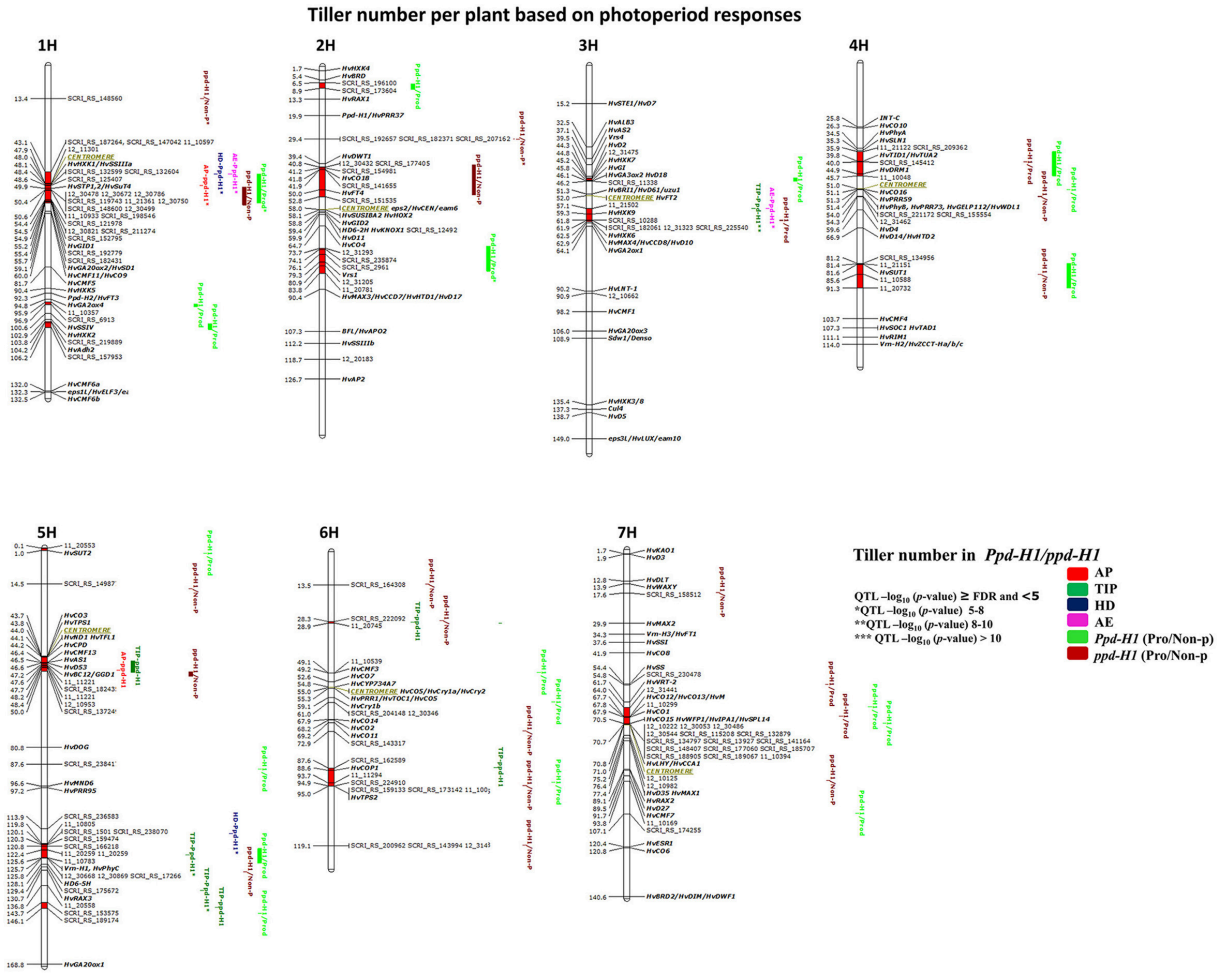
**Genetically anchored position of highly associated QTL for tiller number per plant at all barley developmental stages based on photoperiod responses [photoperiod-sensitive (***Ppd-H1***)/reduced photoperiod sensitivity (***ppd-H1***)] groups using 9K SNP markers**. Bold and italicized gene names indicate genetically anchored positions of known heading time and plant stature genes in the Barke × Morex RILs. Associated chromosomal regions are highlighted with different colors according to developmental stages. Red chromosomal areas indicate the range of significantly associated QTL (within confidence interval ±5 cM) which are exceeding FDR level of each developmental stage.

GWAS analysis in both photoperiod response groups, i.e., *Ppd-H1* (95 accessions) and *ppd-H1* (123 accessions), identified 51 marker-trait associations (≥FDR) distributed across 17 chromosomal regions (Figure 5). Most of the associated markers were detected from AE to harvest and 10 QTL appeared to be stage-specific at Hrv for productive and non-productive tillering (Figure 5).

One major association was on 1H (43.1–55.7 cM centromeric region), which included 22 associated markers (≥FDR) and the *GA INSENSITIVE DWARF 1* (*HvGID1*), *HEXOKINASE 1* (*HvHXK1*), *SOLUBLE STARCH SYNTHASE* (*HvSSIIIa*), *HEXOSE TRANSPORTATION 1*, 2/ *SUGAR TRANSPORTER* (*HvSTP1,2*/*HvSuT4*) genes (Figure 5). This region has conflicting effects on tiller number, i.e., a positive effect (enhanced tillering) in the *Ppd-H1* group; whereas the effect was negative (reduced tillering) for the *ppd-H1* allele. We detected one strong group-specific association for non-productive tillering (ppd-H1/Non-P^*^), including three markers on 2H at 29.4 cM, but failed to find known candidate gene close to this QTL. On 4H, we found one significantly associated region at 43.5–45.7 cM [i.e., *TWISTED DWARF 1/TUBULIN ALPHA-2 (HvTID1/HvTUA2)* and *HvDRM1*], which is important for productive tillering in both groups specifically for increasing productive tiller number. We found several significant chromosomal regions without known candidate genes. For instance, group-specific (*Ppd-H1*) associations on 1H (95.9–96.9 and 103.8–106.2 cM) and 2H at 6.5–8.9 cM and 73.7–83.8 cM included the major row-type gene *Vrs1*, which influenced productive tiller number at Hrv (Figure 5). The associated region on chromosome 4H at 54.0–54.3 cM is without candidate gene and important for productive tiller number in *Ppd-H1* and non-productive tillering in the *ppd-H1* group. In addition, we detected putatively novel associations on 5H (143.7–146.1 cM) and 6H (28.3–28.9 cM), which appear important for tillering at different developmental stages. These results clearly demonstrate that using photoperiod responses as a basis for dividing our population is worthwhile to better understand the natural genetic variation of tillering in this germplasm panel.

GWAS analysis in the *ppd-H1* group expresses the importance of heading time genes on tillering. On 2H (40.8–52.8 cM) is an example about heading time genes, including *HvCO18* and *HvFT4*, and the region on 5H (119.8–125.8 cM) covering *Vrn-H1*/*Phy-C*. These findings reinforce that some of the heading time genes may have a pleiotropic effect on tillering at different developmental stages.

By association analysis, we found three overlapping, seemingly sugar-related QTL affecting tiller number. Here, chromosome 3H (57.1–62.5 cM includes *HvHXK9* and *HvHXK6*) showed a major effect in the *Ppd-H1* group [(TIP^**^, and AE^*^ (Figure 5) thereby promoting tillering by +1.7 and +1 tillers, respectively)]; while it also increased productive tillering at Hrv in the *ppd-H1* group. The significant chromosomal region on 4H (81.2–91.3 cM) includes the *SUCROSE TRANSPORTER 1* (*HvSUT1*) gene and had an impact on productive tiller number within *Ppd-H1* and non-productive tillering in *ppd-H1*. The third QTL (i.e., for productive tillering; in the *Ppd*-*H1* group) is located on 5HS close to *HvSUT2*. These observations may hint toward the importance of sugar-related genes on tillering in cereals.

Four strong associations included genes related to plant stature, sugar and heading time. On 5H (43.7–50.0 cM; HvCO3, *TREHALOSE-6-PHOSPHATE SYNTHASE1 (HvTPS1), BRASSINOSTEROID C-23 HYDROXYLASE (HvCPD), NARROW LEAF AND DWARF1/ TERMINAL FLOWER1 (HvND1, TFL1), HvAS1, HvD53, and HvBC12/GGD1*), all of these genes are located in the centromeric region, and hence, it is not clear which gene(s) cause the effect. The same conclusion can be drawn for the region on 7H (64–71 cM; *HvCO12/HvCO13/H; HvCO1, WEALTHY FARMERS PANICLE1/ IDEAL PLANT ARCHITECTURE1/SQUAMOSA PROMOTER BINDING PROTEIN-LIKE14 (HvWFP1/HvIPA1/HvSPL14); LATE ELONGATED HYPOCOTYL/CIRCADIAN CLOCK ASSOCIATED1 (HvLHY/HvCCA1)* and similarly on 3H (44.3–46.2 cM; HvD2, HvHXK7, HvGI, HvGA3ox2/HvD18). The importance of the latter QTL is that it is group-specific for productive tillering in the Ppd-H1 group. Finally, the QTL on 6H (87.6–95 cM) includes five associated markers close to *CONSTITUTIVELY PHOTOMORPHOGENIC1* (*HvCOP1*, 88.6 cM), and *TREHALOSE-6-PHOSPHATE SYNTHASE2 (HvTPS2)*. GWAS results stratified according to photoperiod response show that tillering is complex and that genetic variation at late-developmental stages is important to understand the genetic factors controlling the formation of productive and non-productive tillers.

### Genetic variation of plant height

**Figure 6:**
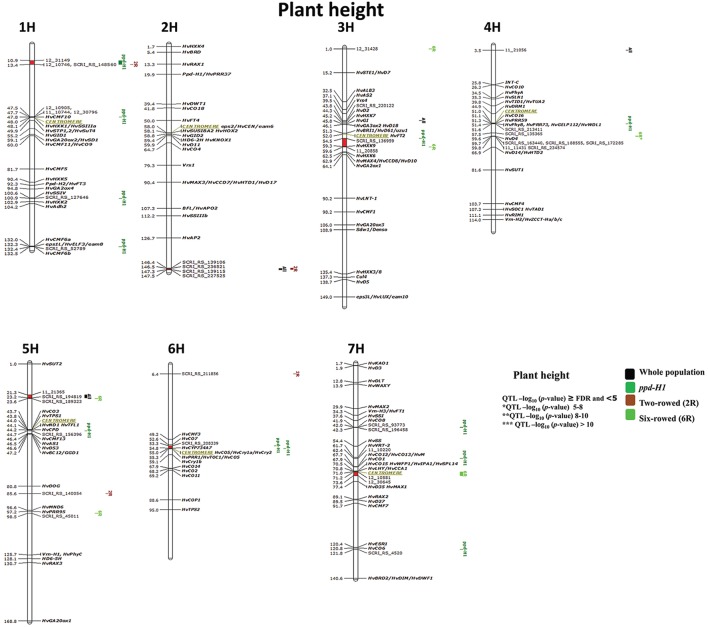
**Genetically anchored position of highly associated QTL for plant height at harvest stage based on spike row-type (two-rowed/six-rowed) and photoperiod responses [photoperiod-sensitive (***Ppd-H1***)/reduced photoperiod sensitivity (***ppd-H1***)] groups using 9K SNP markers**. Bold and italicized gene names indicate genetically anchored positions of known heading time and plant stature genes in the Barke × Morex RILs. Associated chromosomal regions are highlighted with different colors according to group. Red chromosomal areas indicate the range of significantly associated QTL (within confidence interval ±5 cM) which are exceeding FDR level of each group.

There is no clear effect of *vrs1* and *Ppd-H1* on the plant height (Figure S6B), however, we used them to structure the population. GWAS analysis of the entire population detected 10 significant chromosomal regions (Figure 6) with a total of 26 significant marker-trait associations displaying significance (≥FDR). Looking at the genetic variation within groups no marker-trait association was detected for the photoperiod-sensitive group (*Ppd-H1*).

Six significant regions belong to the *ppd-H1* group of which one is without known candidate genes on 1H (10.9–13.4 cM; Figure 6); interestingly all of these six QTL are also closely co-localized with plant height QTL in two- and six-rowed accessions. Two regions very precisely co-localized with putative heading time genes (e.g., on 1H 47.5–48 cM (*HvCMF10*); and on 7H (41.9–42.3 cM) close to *HvCO8*. One significant chromosomal region is a putatively sugar-related QTL on 3H (54.5–59.6 cM) including *HvHXK9*. While two other interesting associations were found around the centromeric region on 4H and 6H including several associated candidate genes for plant height and heading time.

Four marker-trait associations were detected in the subpopulation of two-rowed barley; two of which were single-marker-trait associations (Figure 6; 5H 85.6 and 6H 6.4 cM) and two were located in significant chromosomal regions. The two-rowed-specific QTL on 1H (10.9–13.4 cM) is shared with a *ppd-H1*-specific QTL, while the second QTL located at the end of 2H (146.4–147.5 cM) is overlapping with associations from the whole collection (i.e., ALL; Figure 6). These QTL appeared to be novel without known candidate genes associated with them, indicating that this study is able to reveal potentially new plant height QTL in two-rowed barley.

In six-rowed barley accessions, six marker-trait associations were detected. Two were single-marker-trait associations (Figure 6; 3H 1.0 cM and 5H 95.5 cM); while from the remaining four QTL, one lacks any candidate genes (putatively novel QTL) on 5H (21.3–23.6 cM). The QTL on 3H (54.5–59.6 cM) includes *HvHXK9* and was shared (*ppd-H1* group). The strongest association for plant height was located on 4H between 59.6–59.8 cM^*^, which is co-located with *HvD4*, and supported by 10 markers reducing plant height in six-rowed accessions by 7 cm. Another interesting six-rowed specific association is located in the centromeric region on 7H (71.2–73.6 cM).

GWAS analysis for plant height using different population structures revealed an association with sugar-related and heading time genes on plant height. Nonetheless, we found putatively novel QTL regions, which certainly require further validation work.

## Discussion

### The significance of the experimental approach

Analysis of tillering at different pre-anthesis developmental stages provided an unprecedented overview on the natural variation of tiller outgrowth in our worldwide barley collection. This approach appears to be helpful to better understand genetic factors controlling tillering in cereals. Previous tillering studies in barley were mainly conducted under field conditions to associate the final number of tillers at harvest with yield or developmental stages like HD (Borras et al., 2009; Alqudah and Schnurbusch, 2014). High broad-sense heritability values for plant stature-related traits were obtained in comparison with previous studies (Rasmusson, 1987; Borras et al., 2009; Pauli et al., 2014), most likely because of accurate phenotyping following a single-plant phenotyping strategy at different developmental stages under controlled GH conditions (Alqudah and Schnurbusch, 2014, 2015; Alqudah et al., 2014). Therefore, the power of the current GWAS to detect associated loci was increased compared with previous field studies of the same germplasm (e.g., Pasam et al., 2012), demonstrating that GH conditions are appropriate for studying plant stature-related traits.

Spike row-type classes in barley (two- and six-rowed) were found as one of the major determinants of population structure in most barley GWAS analyses (Pasam et al., 2012; Pauli et al., 2014); however, we subdivided our population based on *Ppd-H1* alleles and row-type classes and hence were able to detect a rich source of genetic variation for plant stature traits. The solidity of the found marker-trait associations (FDR) approach, e.g., also used by Pauli et al. (2014), in combination with the latest version of the barley physical map enabled us to clearly locate genetic marker positions and associate detected QTL with candidate gene(s). This approach has the potential to create novel, hypothesis-driven research questions but similarly may provide a glimpse into ontogenetic traits, which are associated with specific gene classes, families, hormones, or metabolic pathways.

### QTL for tiller number per plant

Several putatively novel candidate regions were associated with tillering at different developmental stages based on row-type and/or photoperiod response groups. For instance, in the present study we detected putatively novel QTL without known candidates for productive tiller number in the *Ppd-H1* group. The QTL on 1H (95.9–96.9 cM), 2H (6.5–8.9 cM) and 5H (143.7–146.1 cM) showed that there may be an opportunity to genetically optimize yield through increased productive tillering. Interestingly, the QTL on 2H at 6.5–8.9 cM is close to *HvBRD*, known as an important regulator of barley plant stature traits (Dockter et al., 2014), suggesting that this gene could be a putative candidate for controlling productive tiller number, too. Most of the putatively novel QTL appeared at earlier developmental stages in two-rowed barley, which are mostly carrying the *ppd-H1* allele, showing delayed development and thus may produce more tillers. Functional analysis of these novel genomic regions will help to expand our knowledge about tillering in cereals.

The two-rowed group exhibited a more complex genetic make-up for tillering than six-rowed types. The effect of the row-type gene *Vrs1* on tillering at early developmental stages became evident after studying *vrs1* mutants. Here, wild-type plants had significantly more tillers than mutants, which is in agreement with recently published results obtained by Liller et al. (2015). It is known that *Vrs1* determines spike row-type (Komatsuda et al., 2007). Due to its known role as a negative regulator of lateral spikelet fertility in the spike, one might assume that wild-type *Vrs1* also negatively affects tillering; but this was evidentially not the case in our wild-type/mutant analyses and GWAS panel. In fact, lateral spikelet abortion of the spike, but increased tiller number in two-rowed types, is most likely explainable as a negative pleiotropic effect of *Vrs1*, whereby grain setting potential is compensated through tillering; or vice-versa for six-rowed types.

Accessions with delayed heading time (those carrying the *ppd-H1* allele) showed a more complex genetic constitution for tillering, possibly due to longer pre-anthesis phase durations and more non-productive tillering than early heading accessions (*Ppd-H1*). This observation may also reinforce our previous findings that the pre-anthesis period is critical for tiller development and any tiller developed after heading might not develop productive spikes.

Results obtained for hormone-related QTL affecting tillering showed associations especially at Hrv. For instance, expression of *DRM1-like* in wheat is known to be associated with tiller bud dormancy in a *tiller inhibition* (*tin*) mutant (Kebrom et al., 2012). The *HvDRM1* region (4H, 44.9 cM) appeared in six-rowed and photoperiod sensitive groups, suggesting that allelic variation at this chromosomal region is crucial for producing less but mainly productive tillers. Similar conclusions can be drawn for the chromosomal region around *HvTID1/HvTUA2* (4H, 39.8 cM), which is known to control plant stature through changing the number of cells in the shoot apical meristem in rice (Sunohara et al., 2009). In contrast to the *HvDRM1* region, associations close to *BRASSINOSTEROID DEFICIENT DWARF2/ DIMINUTO, DWARF1 (HvBRD2/HvDIM/HvDWF1*; i.e., 7H, 140.6 cM) lead to produce non-productive tillers possibly due to trade-offs with other plant stature traits. These results indicate potential loci controlling tiller number that can be utilized for future breeding programs.

Obtained association signals show for the first time a genetic association for a potential role of sugar-related genes in tillering of barley. In accordance with recent findings in sorghum, sugar is one of the major key regulators of axillary bud outgrowth (Kebrom and Mullet, 2015). Three putatively sugar-related QTL were found to be associated with *HEXOKINASE* and *SUCROSE TRANSPORTER* genes reinforcing the hypothesis about the importance of sugars in tillering. Hexokinases were characterized in rice as being important for sugar phosphorylation, sugar sensing, and signaling (Cho et al., 2006). Recently, it was shown that sucrose plays a key role during shoot branching in wheat and pea (Kebrom et al., 2012; Mason et al., 2014). Moreover, the expression of sucrose-inducible genes was down regulated in dormant buds of the *tin* mutant of wheat (Kebrom et al., 2012). In summary, our association analysis suggests that there is a tight linkage between sugar-related genes and productive tiller number that predominantly appears in accessions carrying *Ppd-H1* alleles. Future studies should investigate the mechanisms of how sugar-related genes influence tillering and plant height.

Marker-trait associations explored the importance of putative heading time genes particularly those carrying *CCT* [*CO, CO-LIKE, TIMING OF CAB1 (TOC1)*] domain and B-box domains (*CO-like* genes) in the natural variation of tillering. These findings imply that *CO-like* genes might also be involved in tillering; however, more genetic analyses are required to elucidate their role and expand our current knowledge about these genes. Notably, the region around *BFL* (2H, 107.3 cM) was strongly associated with several SNPs in the two-rowed group, suggesting that this region has an important role in tillering in addition to regulating phase duration (Alqudah et al., 2014). Here, significant effects were found for markers co-locating with *BFL* thereby reducing tiller number by one tiller per plant. Further characterization of this gene is necessary to evaluate its importance in barley plant stature.

Another interesting association was found in the chromosomal region that includes *COP1* (6H, 88.6 cM) especially when *Ppd-H1* alleles were less active (i.e., *ppd-H1*, more tillers). Arabidopsis *COP1* regulates photomorphogenesis in seedlings and it also has pleiotropic phenotypes during late developmental stages (Nakagawa and Komeda, 2004). *HvCOP1* appears to be a late heading time gene (Alqudah et al., 2014) which likely promotes tillering in the late heading *ppd-H1* group. Taken together, allelic variation around *HvCOP1* appears as the first report for temperate cereals that this gene affects tillering possibly through controlling vegetative-to-generative phase-transition.

Two strong associations were found in the centromeric regions of 5 and 7H with tight linkage to hormone and heading time genes in photoperiod response groups; due to the uncertainty of marker orders in these regions, drawing final conclusions require more genetic evidences.

Interestingly, we found that improved cultivars produced more tillers likely as an output of breeding programs. This feature appeared in many EU cultivars, which are mostly two-rowed, possess the late *ppd-H1* allele and thus produce more tillers that are non-productive. Manipulating tiller number genetically by decreasing non-productive tillering and/or increasing productive tiller number will be a challenge for breeders to maximize yield. Using QTL analysis in wheat, Xie et al. (2016) proposed that large genetic variation in tillering is advantageous to select for higher tillering capacity and survival thereby producing more fertile tillers that then may contribute to higher grain yield. Considering all of our findings from tillering, one can conclude that natural variation of tillering is under a complex genetic regulation. Our findings reinforce that pleiotropic gene actions do exist for tiller number, for example in case of *Vrs1*. Here, we set out to obtain a broad overview of the genetic factors that influence tillering in barley while follow-up work in other cereals will gain value-added information in this context.

### QTL for plant height

In this study, we found three putatively novel plant height QTL (1H, 10.9–13.4; 2H, 146.4–147.5; 5H, 21.3–23.6) which were not reported in previous GWAS analyses such as Pasam et al. (2012) and Pauli et al. (2014) or bi-parental mapping studies (Wang et al., 2014) conducted under field conditions. In-depth genetic analyses of these important QTL are worthwhile targets to improve lodging resistance and subsequently yield.

In our germplasm panel, natural genetic variation for plant height was genetically less complex than for tillering, most likely due to the low variation in plant height as was reported by Pasam et al. (2012). The *vrs1* mutant analysis suggests that *Vrs1* also regulates plant height in addition to lateral spikelet/floret development and tillering. Associations close to *HvD4* predominantly appeared in six-rowed accessions. This gene is known to impact plant height in rice, where mutants show mild semi dwarfism due to defects in brassinosteroid biosynthesis (Sakamoto et al., 2006). Thus, variation for plant height in our collection could be attributed to brassinosteroid deficiency.

Interestingly, our GWAS analysis suggests that sugar-related genes are involved in regulating plant height. For instance, associations at *HvHXK9* (3H, 59.3 cM) and allelic variation around this gene appear as the first report for temperate cereals that sugar-related genes are possibly also important for plant height. Clearly, further molecular and genetic investigations are required in order to reveal the role of sugars in plant height.

Putative heading time genes, such as *HvCMF10* and *HvCO8*, were closely associated with plant height. An effect of heading time genes like *Ppd-H1* and *Flt-2L* on plant height was already reported in previous studies (Karsai et al., 1999; Chen et al., 2009). Interestingly, the centromeric region around Os*WFP1*/Os*IPA1*/Os*SPL14* (7H, 70.5 cM) was associated with plant height in six-rowed barley; while this gene regulates plant architecture, including plant height in rice (Jiao et al., 2010; Miura et al., 2010), we cannot exclude the effect of other closely linked genes, such as *HvLHY* and *HvCCA1* (7H, 70.8 cM). Similar conclusion can be postulated for genes in the centromeric regions of 4 and 6H. Thus, further genetic and functional analyses of these regions may reveal the importance of these genes in barley plant height research. Thus, these findings provide an overview about the genetic factors influencing plant height in a diverse spring barley collection, including several novel QTL and newly identified genes.

## Conclusion

In the context of plant architecture, we found substantial differences for tillering and plant height in our barley worldwide collection. The analysis once more demonstrated the power of the GWAS approach for identifying putative candidate genes and improving plant architecture. Several physically anchored and co-locating chromosomal segments harboring known plant stature-related phytohormone metabolism and signaling genes in addition to sugar-related genes were identified. Based on GWAS results, a link between the genetic control of row-type, heading time, tillering, and plant height in barley was established. Our findings suggest that considering sugar-related genes seems very promising for future barley plant architecture works. Further investigation to confirm these associations, i.e., further functional validation analysis of candidate associations found in this work is imperatively required to better understand the genetic control of plant architecture in cereals.

## Author contributions

Conceived the project: TS. Designed and performed the experiments: AA, RK, GW, TS. Analyzed the data: AA, TS. Contributed reagents/materials/analysis tools: AG. Wrote the paper: AA, TS with contributions from all co-authors.

### Conflict of interest statement

The authors declare that the research was conducted in the absence of any commercial or financial relationships that could be construed as a potential conflict of interest.
