# Supplementary material for: The Genetic Architecture of Barley Plant Stature
Source: Front Genet. 2016 Jun 24;7:117. doi: 10.3389/fgene.2016.00117 (PMC4919324; doi:10.3389/fgene.2016.00117)
Supplement: Supplementary file 1 [file Table1.docx]

**The genetic architecture of barley plant stature**

**Frontiers in Genetics 7**

DOI: [10.3389/fgene.2016.00117](http://journal.frontiersin.org/article/10.3389/fgene.2016.00117/abstract)

Ahmad M. Alqudah^1🖂^; Ravi Koppolu^1^; Gizaw M. Wolde^1^; Andreas Graner^2^; Thorsten Schnurbusch^1🖂^

^1^HEISENBERG-Research Group Plant Architecture,

^2^Research Group Genome Diversity,

Leibniz Institute of Plant Genetics and Crop Plant Research (IPK),

Corrensstrasse 3, OT Gatersleben, D-06466 Stadt Seeland, Germany

^🖂^Corresponding authors:

Ahmad M. Alqudah,

Tel: +49-39482-5826, email: [alqudah@ipk-gatersleben.de](mailto:alqudah@ipk-gatersleben.de)

PD Dr. Thorsten Schnurbusch,

Tel: +49-39482-5341, Fax: +49-39482-5595, email: [thor@ipk-gatersleben.de](mailto:thor@ipk-gatersleben.de)

HEISENBERG-Research Group Plant Architecture

Leibniz Institute of Plant Genetics and Crop Plant Research (IPK)

Corrensstrasse 3, OT Gatersleben, D-06466 Stadt Seeland, Germany

**Table S1**: Spike row-type, origin and photoperiod-sensitivity of spring barleys accessions.

| Origin‡ |  | Photoperiod-sensitive  (*Ppd-H1*) | |  | | Reduced photoperiod sensitivity (*ppd-H1*) | | | |  | | Total | | |  |
| --- | --- | --- | --- | --- | --- | --- | --- | --- | --- | --- | --- | --- | --- | --- | --- |
|  |  | **Two-rowed** | **Six-rowed** | |  | | **Two-rowed** | | **Six-rowed** | |  | | |  | |
| WANA |  | 12 | 21 | |  | | 11 | | 1 | |  | | | 45 | |
| EU |  | 10 | 6 | |  | | 80 | | 12 | |  | | | 108 | |
| EA |  | 0 | 28 | |  | | 2 | | 6 | |  | | | 36 | |
| AM |  | 6 | 12 | |  | | 4 | | 7 | |  | | | 29 | |
| Total |  | **28** | **67** | |  | | **97** | | **26** | |  | | | **218** | |
|  |  | **95** | | | | | | **123** | | | | |  |  |  |

‡ WANA: West Asia and North Africa, EU: Europe, EA: East Asia, AM, Americas.
