# Supplementary material for: The Genetic Architecture of Barley Plant Stature
Source: Front Genet. 2016 Jun 24;7:117. doi: 10.3389/fgene.2016.00117 (PMC4919324; doi:10.3389/fgene.2016.00117)
Supplement: Supplementary file 3 [file Table3.docx]

**Table S3**: GenBank accession number for known heading time and plant stature/architecture candidate genes with their POPSEQ genetic position and significantly associated markers (POPSEQ position in cM).

**The genetic architecture of barley plant stature**

**Frontiers in Genetics 7**

DOI: [10.3389/fgene.2016.00117](http://journal.frontiersin.org/article/10.3389/fgene.2016.00117/abstract)

Ahmad M. Alqudah^1🖂^; Ravi Koppolu^1^; Gizaw M. Wolde^1^; Andreas Graner^2^; Thorsten Schnurbusch^1🖂^

^1^HEISENBERG-Research Group Plant Architecture,

^2^Research Group Genome Diversity,

Leibniz Institute of Plant Genetics and Crop Plant Research (IPK),

Corrensstrasse 3, OT Gatersleben, D-06466 Stadt Seeland, Germany

^🖂^Corresponding authors:

Ahmad M. Alqudah,

Tel: +49-39482-5826, email: [alqudah@ipk-gatersleben.de](mailto:alqudah@ipk-gatersleben.de)

PD Dr. Thorsten Schnurbusch,

Tel: +49-39482-5341, Fax: +49-39482-5595, email: [thor@ipk-gatersleben.de](mailto:thor@ipk-gatersleben.de)

HEISENBERG-Research Group Plant Architecture

Leibniz Institute of Plant Genetics and Crop Plant Research (IPK)

Corrensstrasse 3, OT Gatersleben, D-06466 Stadt Seeland, Germany

| **Chr.** | **Gene** | **GenBank accession number** | **Barley**  **High Conf.**  **gene** | **Reference** | **cM**  **(POP SEQ)** | **Contig identifier** | **Significantly associated SNP marker ≥FDR (cM POPSEQ)** | | |
| --- | --- | --- | --- | --- | --- | --- | --- | --- | --- |
|  |  |  |  |  |  |  | **Tillering** | | **Plant height** |
|  |  |  |  |  |  |  | *Ppd-H1/ppd-H1* | 2R/6R |  |
|  |  |  |  |  |  |  |  |  |  |
| 1H | *HEXOKINASE 1 (HvHXK1),*  *SOLUBLE STARCH SYNTHASE (HvSSIIIa)* | [HM037127.1](http://www.ncbi.nlm.nih.gov/nuccore/327555162?report=fasta)  [FN179377.1](http://www.ncbi.nlm.nih.gov/nuccore/FN179377.1) | [MLOC_57896.1](http://apex.ipk-gatersleben.de/apex/f?p=284:20:::NO::P20_GENE_NAME:MLOC_57896.1) | ([Cho et al., 2006](#_ENREF_6)),  ([Radchuk et al., 2009](#_ENREF_34)) | 48.08 | morex_contig_42192 CAJW010042192,  morex_contig_41334 CAJW010041334 | (Ppd-H1, ppd-H1 SCRI_RS_132599;SCRI_RS_132604; SCRI_RS_125407 ( HD , AE, Pro, Non-P) 48.4- 48.6) |  |  |
| 1H | *HEXOSE*  *TRANSPORTATION1, 2/SUGAR TRANSPORTER (HvSTP1,2/HvSuT4)* | [AJ534445.1](http://www.ncbi.nlm.nih.gov/nuccore/26986185?report=fasta)  [AJ534446](http://www.ncbi.nlm.nih.gov/nuccore/26986187?report=fasta) | [MLOC_53885.1](http://apex.ipk-gatersleben.de/apex/f?p=284:20:::NO::P20_GENE_NAME:MLOC_53885.1) | ([Weschke et al., 2003](#_ENREF_45)) | 49.85 | morex_contig_38718 CAJW010038718 | (Ppd-H1, ppd-H1  12_30478  12_30672  12_30786  SCRI_RS_119743  11_21361  12_30750  SCRI_RS_148600  12_30499  11_10933  SCRI_RS_198546  (Pro, Non-P) 50.4-50.6) |  |  |
| 1H | *GIBBERELLIN INSENSITIVE DWARF1 (HvGID1)* | [AK074026](http://getentry.ddbj.nig.ac.jp/getentry/ddbj/AK074026?filetype=html) | [AK356665](http://apex.ipk-gatersleben.de/apex/f?p=284:20:::NO::P20_GENE_NAME:AK356665) | <http://rice.plantbiology.msu.edu/cgi-bin/ORF_infopage.cgi?orf=LOC_Os05g33730.1> | 55.52 | morex_contig_137029 CAJW010137029 | (ppd-H1, SCRI_RS_21483 (Hrv; 55.5) |  |  |
| 1H | *GIBBERELLIN 20 OXIDASE 2/ SEMIDWARF 1 (HvGA20ox2/HvSD1)* | [CT834906](http://getentry.ddbj.nig.ac.jp/getentry/ddbj/CT834906?filetype=html) | [MLOC_56462.1](http://apex.ipk-gatersleben.de/apex/f?p=284:20:::NO::P20_GENE_NAME:MLOC_56462.1) | ([Yang et al., 2009](#_ENREF_47)) ([Sakamoto et al., 2004](#_ENREF_36)) | 59.13 | morex_contig_244138 CAJW010244138 |  |  |  |
| 1H | *HEXOKINASE 5 (HvHXK5)* | [HM037131.1](http://www.ncbi.nlm.nih.gov/nuccore/327555170?report=fasta) | [MLOC_53317.2](http://apex.ipk-gatersleben.de/apex/f?p=284:20:::NO::P20_GENE_NAME:MLOC_53317.2) | ([Mangelsen et al., 2011](#_ENREF_29)) | 90.43 | morex_contig_38242 CAJW010038242 |  |  |  |
| 1H | *GIBBERELLIN 20 OXIDASE 4 (HvGA2ox4)* | [AY551432.1](http://www.ncbi.nlm.nih.gov/nuccore/AY551432.1) | [MLOC_13981.1](http://apex.ipk-gatersleben.de/apex/f?p=284:20:::NO::P20_GENE_NAME:MLOC_13981.1) | ([Spielmeyer et al., 2004](#_ENREF_40)) | 94.75 | morex_contig_1566970 CAJW011566970 | (ppd-H1, SCRI_RS_136856 (Hrv); 94.90) |  |  |
| 1H | *SOLUBLE STARCH SYNTHASE (HvSSIV)* | [FN179379.1](http://www.ncbi.nlm.nih.gov/nuccore/229610860?report=fasta) | [AK355447](http://apex.ipk-gatersleben.de/apex/f?p=284:20:::NO::P20_GENE_NAME:AK355447) | ([Radchuk et al., 2009](#_ENREF_34)) | 100.56 | morex_contig_48282 CAJW010048282 |  |  | Ppd-H1 SCRI_RS_127646 100.9 |
| 1H | *HEXOKINASE 2 (HvHXK2)* | [HM037128.1](http://www.ncbi.nlm.nih.gov/nuccore/327555164?report=fasta) | [MLOC_10933.1](http://apex.ipk-gatersleben.de/apex/f?p=284:20:::NO::P20_GENE_NAME:MLOC_10933.1) | ([Mangelsen et al., 2011](#_ENREF_29)) | 102.94 | morex_contig_1560054 CAJW011560054 | Ppd-H1, Pro SCRI_RS_219889 103.8 c1 |  |  |
| 2H | *HEXOKINASE 4 (HvHXK4)* | HM037130.1 | [AK370117](http://apex.ipk-gatersleben.de/apex/f?p=284:20:::NO::P20_GENE_NAME:AK370117) | ([Cho et al., 2006](#_ENREF_6)) | 1.68 | morex_contig_267414 CAJW010267414 |  |  |  |
| 2H | *BRASSINOSTEROID-6-OXIDASE HvBRD)* | [KF318308.1](http://www.ncbi.nlm.nih.gov/nuccore/552350664) | [AK372445](http://apex.ipk-gatersleben.de/apex/f?p=284:20:::NO::P20_GENE_NAME:AK372445) | ([Dockter et al., 2014](#_ENREF_12)) | 5.38 | morex_contig_244330 CAJW010244330 | (ppd-H1,  11_20372  SCRI_RS_13476  12_10592 (Hrv); 6.45-7.44) | (2R,  SCRI_RS_133377  SCRI_RS_169758  SCRI_RS_13476  11_21377  (AP); 6.58-7.43) |  |
| 2H | *REGULATOR OF AXILLARY MERISTEMS1 (HvRAX1)* | [NP_197691.1](http://www.ncbi.nlm.nih.gov/protein/15237193) |  | ([Lin et al., 1999](#_ENREF_28)) | 13.31 | morex_contig_136785 |  |  |  |
| 2H | *PHOTOPERIOD RESPONSE LOCUS1 (PpdH1)* | [AY970701.1](http://www.ncbi.nlm.nih.gov/nuccore/AY970701.1) | [MLOC_81154.10](http://apex.ipk-gatersleben.de/apex/f?p=284:20:::NO::P20_GENE_NAME:MLOC_81154.10) | ([Comadran et al., 2012](#_ENREF_8)) | 19.9 | morex_contig_94710 CAJW010094710 |  | (2R, SCRI_RS_210172 (AP, TIP, HD, AE), SCRI_RS_233272 (AP,TIP, HD, AE), SCRI_RS_153798 (AP, TIP), SCRI_RS_170337 (AP, TIP, HD, Hrv), 11_21015 (AP, TIP, HD, Hrv), SCRI_RS_143250 (TIP), BK_14 (AP, TIP, HD, Hrv), BK_16 (AP, TIP, HD, Hrv); 18.9-19.9) |  |
| 2H | *HvFT4* | [DQ411320.1](http://webblast.ipk-gatersleben.de/barley/blastresult.php?jobid=140120761128&opt=none) | [MLOC_74854.1](http://apex.ipk-gatersleben.de/apex/f?p=284:20:::NO::P20_GENE_NAME:MLOC_74854.1) | ([Faure et al., 2007](#_ENREF_13)) | 50.04 | morex_contig_6666 CAJW010006666 |  | (2R, 11_21005, SCRI_RS_158649 (TIP, AE); 50.9 |  |
| 2H | *eps2/HvCEN/eam6* | [JX844786.1](http://www.ncbi.nlm.nih.gov/nuccore/410443466?report=fasta) | [MLOC_44160.1](http://apex.ipk-gatersleben.de/apex/f?p=284:20:::NO::P20_GENE_NAME:MLOC_44160.1) | ([Comadran et al., 2012](#_ENREF_8)) | 58.00 | morex_contig_274284 CAJW010274284 | (ppd-H1,  11_10329  SCRI_RS_151430  SCRI_RS_141874  (Hrv); 57-58.07) | (2R, SCRI_RS_135633,  11_10624,  SCRI_RS_230984  11_10436,  11_21110,  11_10909,  11_20374 (AP); 57.43- 59.34) |  |
| 2H | *HOMEODOMAIN LEUCINE ZIPPER BOX (HvHOX2)* | [AB490234.1](http://www.ncbi.nlm.nih.gov/nuccore/AB490234.1) | [MLOC_77488.1](http://apex.ipk-gatersleben.de/apex/f?p=284:20:::NO::P20_GENE_NAME:MLOC_77488.1) | ([Sakuma et al., 2010](#_ENREF_37)) | 58.05 | morex_contig_7586 CAJW010007586 | (ppd-H1,  11_10329  SCRI_RS_151430  SCRI_RS_141874  (Hrv, non-P); 57-58.07) | (2R, SCRI_RS_135633  11_10624,  SCRI_RS_230984  11_10436,  11_21110,  11_10909,  11_20374 (AP); 57.43- 59.34) |  |
| 2H | *SUGAR SIGNALLING IN BARLEY 2 (HvSUSIBA2)* | [AY323206.1](http://www.ncbi.nlm.nih.gov/nuccore/34329336?report=fasta) | [AK369730](http://apex.ipk-gatersleben.de/apex/f?p=284:20:::NO::P20_GENE_NAME:AK369730) | ([Sun et al., 2003](#_ENREF_41)) | 58.05 | morex_contig_206649 CAJW010206649 | (ppd-H1,  11_10329  SCRI_RS_151430  SCRI_RS_141874  (Hrv, non-P); 57-58.07) | (2R, SCRI_RS_135633  11_10624,  SCRI_RS_230984  11_10436,  11_21110,  11_10909,  11_20374 (AP); 57.43- 59.34) |  |
| 2H | *GIBBERELLIN-INSENSITIVE DWARF 2 (HvGID2)* | [AK066506](http://getentry.ddbj.nig.ac.jp/getentry/ddbj/AK066506?filetype=html) | [MLOC_61457.1](http://apex.ipk-gatersleben.de/apex/f?p=284:20:::NO::P20_GENE_NAME:MLOC_61457.1) | <http://rice.plantbiology.msu.edu/cgi-bin/ORF_infopage.cgi?orf=LOC_Os02g36974.1> | 58.78 | morex_contig_41142 |  | (2R, SCRI_RS_135633  11_10624,  SCRI_RS_230984  11_10436,  11_21110,  11_10909,  11_20374 (AP); 57.43- 59.34)  (Til, ppd-H1,  SCRI_RS_137710 (Hrv); 58.78) |  |
| 2H | *KNOTTED1-LIKE HOMEOBOX1 (HvKNOX1)* | [NP_001149876.1](http://www.ncbi.nlm.nih.gov/protein/NP_001149876.1) |  | ([Alexandrov et al., 2009](#_ENREF_1)) | 59.41 | morex_contig_1561605 | (ppd-H1,  SCRI_RS_12492  (Hrv); 59.35) | (2R, SCRI_RS_135633,  11_10624,  SCRI_RS_230984,  11_10436,  11_21110,  11_10909,  11_20374 (AP); 57.43- 59.34) |  |
| 2H | *HD6-2H* | [DQ157464.1](http://webblast.ipk-gatersleben.de/barley/blastresult.php?jobid=140125780669&opt=none) | [MLOC_55943.1](http://apex.ipk-gatersleben.de/apex/f?p=284:20:::NO::P20_GENE_NAME:MLOC_55943.1) | <http://kasetsartjournal.ku.ac.th/kuj_files/2008/A080402133514.pdf> | 59.41 | morex_contig_1567582 CAJW011567582 | (ppd-H1,  SCRI_RS_12492  (Hrv); 59.35) | (2R, SCRI_RS_135633  11_10624,  SCRI_RS_230984  11_10436,  11_21110,  11_10909,  11_20374 (AP); 57.43- 59.34) |  |
| 2H | *DWARF 11 (HvD11), CYTOCHROME P450 724B1* | [AB158759.1](http://www.ncbi.nlm.nih.gov/nuccore/50058151) | [AK371371](http://apex.ipk-gatersleben.de/apex/f?p=284:20:::NO::P20_GENE_NAME:AK371371) | ([Tanabe et al., 2005](#_ENREF_43)) | 59.91 | morex_contig_45000 CAJW010045000 | (ppd-H1,  11_20251  SCRI_RS_4802 (Hrv); 59.90-60.80)) | (2R, SCRI_RS_135633  11_10624,  SCRI_RS_230984  11_10436,  11_21110,  11_10909,  11_20374 (AP); 57.43- 59.34 |  |
| 2H | *HvCO4* | [AF490474](http://webblast.ipk-gatersleben.de/barley/blastresult.php?jobid=140120756094&opt=none) | [AK355626](http://apex.ipk-gatersleben.de/apex/f?p=284:20:::NO::P20_GENE_NAME:AK355626) | ([Griffiths et al., 2003](#_ENREF_14)) | 64.73 | morex_contig_161048 CAJW010161048 | (ppd-H1,  12_10717 (Hrv); 64.59) | (2R, SCRI_RS_163975 (AP); 64.44. |  |
| 2H | *SIX-ROWED SPIKE 1 (Vrs1)* | [AB259782.1](http://www.ncbi.nlm.nih.gov/nuccore/AB259782.1) | [MLOC_77488.1](http://apex.ipk-gatersleben.de/apex/f?p=284:20:::NO::P20_GENE_NAME:MLOC_77488.1) | ([Komatsuda et al., 2007](#_ENREF_24)) | 79.30 | morex_contig_135757 CAJW010135757 | (ppd-H1, 12_30896 (Hrv); 79.3) | (2R, 12_30896 (Hrv); 79.3) |  |
| 2H | *MORE AXILLARY BRANCHES 3/ CAROTENOID CLEAVAGE DIOXYGENASE 7/ HIGH-TILLERING DWARF 1/ DWARF 17 (HvMAX3/HvCCD7/HvHTD1/HvD17)* | [FJ957945.1](http://www.ncbi.nlm.nih.gov/nuccore/237908816) | [MLOC_55474.1](http://apex.ipk-gatersleben.de/apex/f?p=284:20:::NO::P20_GENE_NAME:MLOC_55474.1) | ([Booker et al., 2004](#_ENREF_3))  <http://rice.plantbiology.msu.edu/cgi-bin/ORF_infopage.cgi?orf=LOC_Os04g46470.1> | 90.43 | morex_contig_40001 CAJW010040001 |  |  |  |
| 2H | *BARLEY FLORICAULA LEAFY/ ABERRANT PANICLE ORGANIZATION2 (BFL/HvAPO2)* | [AB005620.1](http://webblast.ipk-gatersleben.de/barley/blastresult.php?jobid=140120769868&opt=none) | [MLOC_14305.1](http://apex.ipk-gatersleben.de/apex/f?p=284:20:::NO::P20_GENE_NAME:MLOC_14305.1) | ([Kyozuka et al., 1998](#_ENREF_26)) | 107.36 | morex_contig_1567741 CAJW011567741 | (ppd-H1, SCRI_RS_223885 (Hrv); 106.40) | (2R, 11_10731,  12_31402,  11_20064 (TIP); 107.36-107.86) |  |
| 2H | *SOLUBLE STARCH SYNTHASE (HvSSIIIb)* | [FN179378.1](http://www.ncbi.nlm.nih.gov/nuccore/229610858?report=fasta) | [MLOC_54759.1](http://apex.ipk-gatersleben.de/apex/f?p=284:20:::NO::P20_GENE_NAME:MLOC_54759.1) | ([Radchuk et al., 2009](#_ENREF_34)) | 112.18 | morex_contig_39371 CAJW010039371 |  | (2R, SCRI_RS_154176, 11_10916  SCRI_RS_144379  SCRI_RS_207327 SCRI_RS_142593SCRI_RS_151556, SCRI_RS_183064  12_30598,  12_31264  11_21220  (TIP) 110.2-113.3 |  |
| 2H | *APELATA2/ ZEOCRITON (Ertr67 HvAP2/ Zeo)* | [KC898651](http://webblast.ipk-gatersleben.de/barley/blastresult.php?jobid=140161945454&opt=none#BL_ORD_ID:2785527) |  | ([Houston et al., 2013](#_ENREF_17)) | 127.05 | morex_contig_43451 CAJW010043451 |  |  |  |
| 2H | *DWARF TILLER1 (HvDWT1)* | [BQ466860.1](http://www.ncbi.nlm.nih.gov/nucest/BQ466860.1) | [AK370609](http://apex.ipk-gatersleben.de/apex/f?p=284:20:::NO::P20_GENE_NAME:AK370609) | ([Zhang et al., 2004](#_ENREF_49)) | 36.99 (unpublished data) | morex_contig_96952 |  |  |  |
| 3H | *STEROL METHYLTRANSFERASE 1, DWARF 7 (HvSTE1/HvD7)* | [AK111908](http://getentry.ddbj.nig.ac.jp/getentry/ddbj/AK111908?filetype=html) | [AK371953](http://apex.ipk-gatersleben.de/apex/f?p=284:20:::NO::P20_GENE_NAME:AK371953) | <http://rice.plantbiology.msu.edu/cgi-bin/ORF_infopage.cgi?orf=LOC_Os01g04260.1> | 15.15 | morex_contig_2546814 CAJW012546814 |  |  |  |
| 3H | *ALBINO3 (HvALB3)* | [NP_001189626.1](http://www.ncbi.nlm.nih.gov/protein/334184544) |  | ([Lin et al., 1999](#_ENREF_28)) | 32.48 | morex_contig_7478 |  |  |  |
| 3H | *ASPARAGINE SYNTHETASE 2 (AS2)* | [AY193714.1](http://www.ncbi.nlm.nih.gov/nuccore/28395525) | [AK373732](http://apex.ipk-gatersleben.de/apex/f?p=284:20:::NO::P20_GENE_NAME:AK373732) | ([Møller et al., 2003](#_ENREF_32)) | 37.11 | morex_contig_6705 CAJW010006705 |  |  |  |
| 3H | *SIX-ROWED*  *SPIKE4 (Vrs4)* | [KC854554.1](http://www.ncbi.nlm.nih.gov/nucleotide/KC854554) | [MLOC_61156.1](http://apex.ipk-gatersleben.de/apex/f?p=284:20:::NO::P20_GENE_NAME:MLOC_61156.1) | ([Koppolu et al., 2013](#_ENREF_25)) | 39.51 | morex_contig_2547112 CAJW012547112 |  |  |  |
| 3H | *DWARF 2 (HvD2), CYTOCHROME P450 90D2* | [BT084047](http://getentry.ddbj.nig.ac.jp/getentry/na/BT084047/?filetype=html) | [MLOC_62829.1](http://apex.ipk-gatersleben.de/apex/f?p=284:20:::NO::P20_GENE_NAME:MLOC_62829.1) | ([Soderlund et al., 2009](#_ENREF_39)) | 44.26 | morex_contig_47012 CAJW010047012 | (ppd-H1, SCRI_RS_229693 (Hrv); 44.26) |  |  |
| 3H | *HEXOKINASE 7 (HvHXK7)* | [HM037133.1](http://www.ncbi.nlm.nih.gov/nuccore/327555174?report=fasta) | [MLOC_12451.3](http://apex.ipk-gatersleben.de/apex/f?p=284:20:::NO::P20_GENE_NAME:MLOC_12451.3) | ([Mangelsen et al., 2011](#_ENREF_29)) | 45.82 | morex_contig_1563318 CAJW011563318 | (Ppd-H1, 12_31475 (Prod), 44.8 ) |  |  |
| 3H | *GIBBERELLIN 3 OXIDASE 2 (HvGA3ox2)* | [AY551431.1](http://www.ncbi.nlm.nih.gov/nuccore/49065951) | [MLOC_12855.1](http://apex.ipk-gatersleben.de/apex/f?p=284:20:::NO::P20_GENE_NAME:MLOC_12855.1) | ([Spielmeyer et al., 2004](#_ENREF_40)) | 46.03 | orex_contig_51542 CAJW010051542 | (ppd-H1, 11_20340 (Hrv); 46.03) |  |  |
| 3H | *DWARF 18 (HvD18)* | [AB056519.1](http://www.ncbi.nlm.nih.gov/nuccore/AB056519) | [MLOC_12855.1](http://apex.ipk-gatersleben.de/apex/f?p=284:20:::NO::P20_GENE_NAME:MLOC_12855.1) | ([Itoh et al., 2001](#_ENREF_19))  <http://rice.plantbiology.msu.edu/cgi-bin/ORF_infopage.cgi?orf=LOC_Os01g08220.1> | 46.03 | morex_contig_51542 CAJW010051542 | (ppd-H1, 11_20340 (Hrv); 46.03) |  |  |
| 3H | *BRASSINOSTEROID INSENSITIVE 1*  */*SEMIBRACHYTIC/  *Dwarf61* (*HvBRI1/ uzu1 HvD61/)* | [AB109215.1](http://www.ncbi.nlm.nih.gov/nuccore/AB109215.1) | [MLOC_5176.2](http://apex.ipk-gatersleben.de/apex/f?p=284:20:::NO::P20_GENE_NAME:MLOC_5176.2) | ([Saisho et al., 2004](#_ENREF_35)) | 51.34 | morex_contig_58772 CAJW010058772 | (ppd-H1, SCRI_RS_186341  SCRI_RS_142442,  11_10925, 51.6  12_31214  11_20801  (Hrv); 51.2-51.7) |  |  |
| 3H | *HvFT2* | [DQ297407](http://webblast.ipk-gatersleben.de/barley/blastresult.php?jobid=140120896646&opt=none) | [AK373041](http://apex.ipk-gatersleben.de/apex/f?p=284:20:::NO::P20_GENE_NAME:AK373041) | ([Faure et al., 2007](#_ENREF_13)) | 52.03 | morex_contig_1558556 CAJW011558556 |  |  |  |
| 3H | *HEXOKINASE 9 (HvHXK9)* | [DQ116391.1](http://www.ncbi.nlm.nih.gov/nuccore/73918018?report=fasta) | [MLOC_60691.6](http://apex.ipk-gatersleben.de/apex/f?p=284:20:::NO::P20_GENE_NAME:MLOC_60691.6) | ([Cho et al., 2006](#_ENREF_6)) | 59.27 | morex_contig_44667 CAJW010044667 |  |  |  |
| 3H | *HEXOKINASE* 6 (Hv*HXK6*) | [HM037132.1](http://www.ncbi.nlm.nih.gov/nuccore/327555172?report=fasta) | [MLOC_54094.1](http://apex.ipk-gatersleben.de/apex/f?p=284:20:::NO::P20_GENE_NAME:MLOC_54094.1) | ([Mangelsen et al., 2011](#_ENREF_29)) | 62.53 | morex_contig_38880 CAJW010038880 | (ppd-H1, SCRI_RS_10288  SCRI_RS_182061  12_31323  SCRI_RS_225540 (Prod) 61.8-61.9) |  |  |
| 3H | *MORE AXILLARY BRANCHES 4/ CAROTENOID CLEAVAGE DIOXYGENASE 8 /DWARF 10 (HvMAX4/CHvCD8/ HvD10)* | [FJ957946](http://www.ncbi.nlm.nih.gov/nuccore/FJ957946) | [MLOC_66551.1](http://apex.ipk-gatersleben.de/apex/f?p=284:20:::NO::P20_GENE_NAME:MLOC_66551.1) | ([Guan et al., 2012](#_ENREF_15)) | 62.93 | morex_contig_51744 CAJW010051744 | (ppd-H1,  SCRI_RS_225522  SCRI_RS_220192  (Hrv); 62.39-62.53) |  |  |
| 3H | *GIBBERELLIN 20 OXIDASE 1 (HvGA2ox1)* | [JQ994304.1](http://www.ncbi.nlm.nih.gov/nuccore/JQ994304.1) | [AK364775](http://apex.ipk-gatersleben.de/apex/f?p=284:20:::NO::P20_GENE_NAME:AK364775) | ([Kebrom et al., 2013](#_ENREF_23)) | 64.16 | morex_contig_2550522 CAJW012550522 | (ppd-H1,  SCRI_RS_13778  (Hrv); 64.16) |  |  |
| 3H | *LOW NUMBER OF TILLERS-1 (HvLNT-1)* | [GU722210.1](http://www.ncbi.nlm.nih.gov/nuccore/GU722210.1) | [MLOC_66099.1](http://apex.ipk-gatersleben.de/apex/f?p=284:20:::NO::P20_GENE_NAME:MLOC_66099.1) | ([Dabbert et al., 2010](#_ENREF_10)) | 90.22 | morex_contig_51098 CAJW010051098 |  | (2R, 11_21381 (TIP); 90.15) |  |
| 3H | *HvCMF1* | [JQ791213](http://webblast.ipk-gatersleben.de/barley/blastresult.php?jobid=140120922328&opt=none) |  | ([Cockram et al., 2012](#_ENREF_7)) | 98.22 | morex_contig_43834 CAJW010043834 | (ppd-H1,  12_30375  12_30640  (Hrv); 98.37-98.49) |  |  |
| 3H | *GIBBERELLIN 20 OXIDASE 3 (HvGA20ox3)* | [AY551429.1](http://www.ncbi.nlm.nih.gov/nuccore/49065947) | [MLOC_66389.1](http://apex.ipk-gatersleben.de/apex/f?p=284:20:::NO::P20_GENE_NAME:MLOC_66389.1) | ([Spielmeyer et al., 2004](#_ENREF_40)) | 106.02 | morex_contig_51490 CAJW010051490 | (ppd-H1,  SCRI_RS_150063 (AP); 107.8) | (2R, SCRI_RS_14857 (TIP); 105.2) |  |
| 3H | SEMIDWARF 1 (sdw1/denso) | [AB538269.1](http://apex.ipk-gatersleben.de/apex/f?p=284:24:::NO::P24_MOREX_CONTIG:morex_contig_40861) | [MLOC_56462.1](http://apex.ipk-gatersleben.de/apex/f?p=284:20:::NO::P20_GENE_NAME:MLOC_56462.1) | ([Jia et al., 2009](#_ENREF_20)) | 108.85 | morex_contig_40861 |  |  |  |
| 3H | *HEXOKINASE 3/8 (HvHXK3/8)* | [HM037129.1](http://www.ncbi.nlm.nih.gov/nuccore/327555166?report=fasta) | [AK354713](http://apex.ipk-gatersleben.de/apex/f?p=284:20:::NO::P20_GENE_NAME:AK354713) | ([Cho et al., 2006](#_ENREF_6)) | 135.48 | morex_contig_135038 CAJW010135038 |  |  |  |
| 3H | *semidwarf 1 (sdw1/denso)* | [AB538269.1](http://apex.ipk-gatersleben.de/apex/f?p=284:24:::NO::P24_MOREX_CONTIG:morex_contig_40861) | [MLOC_56462.1](http://apex.ipk-gatersleben.de/apex/f?p=284:20:::NO::P20_GENE_NAME:MLOC_56462.1) | ([Jia et al., 2009](#_ENREF_20)) | 108.85 | morex_contig_40861 |  |  |  |
| 3H | *UNICULME 4 (Cul4)* | [KF151195.1](http://www.ncbi.nlm.nih.gov/nuccore/KF151195.1) | [AK360734](http://apex.ipk-gatersleben.de/apex/f?p=284:20:::NO::P20_GENE_NAME:AK360734) | ([Tavakol et al., 2015](#_ENREF_44)) | 137.33 | morex_contig_40984 CAJW010040984 | (ppd-H1,  12_31500  (Hrv); 137.90) | (Vrs1, SCRI_RS_172357 (HD); 137.74) |  |
| 3H | *DWARF 5 (HvD5)* | [AK100199](http://getentry.ddbj.nig.ac.jp/getentry/ddbj/AK100199?filetype=html) | [MLOC_69179.4](http://apex.ipk-gatersleben.de/apex/f?p=284:20:::NO::P20_GENE_NAME:MLOC_69179.4) |  | 138.7 | morex_contig_56033 |  |  |  |
| 4H | *INTERMEDIUM-C (INT-C)* | [JF904738](http://www.ncbi.nlm.nih.gov/nuccore/JF904738) | [MLOC_70116.1](http://apex.ipk-gatersleben.de/apex/f?p=284:20:::NO::P20_GENE_NAME:MLOC_70116.1) | ([Youssef et al., 2012](#_ENREF_48)) | 25.84 | morex_contig_5747 CAJW010005747 |  |  |  |
| 4H | *WISTED DWARF 1, TUBULIN ALPHA-2 (HvTID1/HvTUA2)* | [AK102560](http://getentry.ddbj.nig.ac.jp/getentry/ddbj/AK102560?filetype=html) | [MLOC_69930.1](http://apex.ipk-gatersleben.de/apex/f?p=284:20:::NO::P20_GENE_NAME:MLOC_69930.1) | <http://rice.plantbiology.msu.edu/cgi-bin/ORF_infopage.cgi?orf=LOC_Os11g14220.1> | 39.80 | morex_contig_57177 CAJW010057177 | (ppd-H1, SCRI_RS_145412 (Hrv); 40) |  |  |
| 4H | *DORMANCY-ASSOCIATED 1 (HvDRM1)* | [DN828863](http://www.ncbi.nlm.nih.gov/nucest/DN828863) | [AK358522](http://apex.ipk-gatersleben.de/apex/f?p=284:20:::NO::P20_GENE_NAME:AK358522) | ([Kebrom et al., 2012](#_ENREF_22)) | 44.90 | morex_contig_246786 CAJW010246786 |  | (2R, 11_20180 (Hrv); 43.48) |  |
| 4H | *GDSL ESTERASE/LIPASE PROTEIN 112, WILTED DWARF AND LETHAL 1 (HvGELP112/HvWDL1)* | [AK067429](http://getentry.ddbj.nig.ac.jp/getentry/na/AK067429/?filetype=html) | [MLOC_14793.1](http://apex.ipk-gatersleben.de/apex/f?p=284:20:::NO::P20_GENE_NAME:MLOC_14793.1) | <http://rice.plantbiology.msu.edu/cgi-bin/ORF_infopage.cgi?orf=LOC_Os11g48070.1> | 51.40 | morex_contig_1569047 CAJW011569047 | (ppd-H1, SCRI_RS_179544  SCRI_RS_200010 (Hrv); 51.13-51.3) |  | (ppd-H1, SCRI_RS_213411; 51.63) |
| 4H | *DWARF 4 (HvD4)* | [AK355174](http://www.ncbi.nlm.nih.gov/nuccore/AK355174) | [AK355174](http://apex.ipk-gatersleben.de/apex/f?p=284:20:::NO::P20_GENE_NAME:AK355174) | ([Dockter et al., 2014](#_ENREF_12)) | 59.63 | morex_contig_61948 CAJW010061948 |  |  | (6R,  11_10639  SCRI_RS_138684  SCRI_RS_196044  SCRI_RS_163440  SCRI_RS_188555  SCRI_RS_234574  11_20924  SCRI_RS_192900  SCRI_RS_172285  11_11431  ; 59.6 -59.8) |
| 4H | *DWARF14/ HIGH-TILLERING DWARF 2 (HvD14/HvHTD2)* | [AK070827](http://getentry.ddbj.nig.ac.jp/getentry/ddbj/AK070827?filetype=html) | [AK363454](http://apex.ipk-gatersleben.de/apex/f?p=284:20:::NO::P20_GENE_NAME:AK363454) | <http://rice.plantbiology.msu.edu/cgi-bin/ORF_infopage.cgi?orf=LOC_Os03g10620.1> | 66.93 | morex_contig_37569 |  |  |  |
| 4H | *SUCROSE TRANSPORTER 1 (HvSUT1)* | [AM055812.1](http://www.ncbi.nlm.nih.gov/nuccore/71890896?report=fasta) | [MLOC_44233.5](http://apex.ipk-gatersleben.de/apex/f?p=284:20:::NO::P20_GENE_NAME:MLOC_44233.5) | ([Sivitz et al., 2005](#_ENREF_38)) | 81.56 | morex_contig_274359 CAJW010274359 | (Ppd-H1, SCRI_RS_134956  11_21151 (Prod) 81.2-81.4) |  |  |
| 4H | *TILLERING AND DWARF 1 (HvTAD1)* | [AK070642](http://getentry.ddbj.nig.ac.jp/getentry/ddbj/AK070642?filetype=html) | [AK370403](http://apex.ipk-gatersleben.de/apex/f?p=284:20:::NO::P20_GENE_NAME:AK370403) | <http://rice.plantbiology.msu.edu/cgi-bin/ORF_infopage.cgi?orf=LOC_Os03g03150.1> | 107.36 | morex_contig_7057 |  |  |  |
| 4H | *RICE DWARF VIRUS MULTIPLICATION 1 (HvRIM1)* | [AB265821.1](http://www.ncbi.nlm.nih.gov/nuccore/AB265821) | [MLOC_74055.1](http://apex.ipk-gatersleben.de/apex/f?p=284:20:::NO::P20_GENE_NAME:MLOC_74055.1) | <http://rice.plantbiology.msu.edu/cgi-bin/ORF_infopage.cgi?orf=LOC_Os03g02800.1> | 111.11 | morex_contig_65072 CAJW010065072 |  |  |  |
| 4H | *SLENDER1(HvSLN1)* | [AF460219.1](http://www.ncbi.nlm.nih.gov/nuccore/AF460219.1) | [AK372064](http://apex.ipk-gatersleben.de/apex/f?p=284:20:::NO::P20_GENE_NAME:AK372064) | ([Chandler et al., 2002](#_ENREF_5)) | 35.31 (Unpublished data) | morex_contig_53931 |  |  |  |
| 5H | *SUCROSE*  *TRANSPORTER 2 (HvSUT2)* | [AJ272308.1](http://www.ncbi.nlm.nih.gov/nuccore/7024412?report=fasta) | [AK362376](http://apex.ipk-gatersleben.de/apex/f?p=284:20:::NO::P20_GENE_NAME:AK362376) | ([Weschke et al., 2000](#_ENREF_46)) | 0.97 | morex_contig_156785 CAJW010156785 | Ppd-H1, 11_20553 (Prod), 0.1) | (2R, SCRI_RS_31797 (AE) 1.7) |  |
| 5H | *TREHALOSE-6-PHOSPHATE SYNTHASE 1 (HvTPS1)* | [HM446020.1](http://www.ncbi.nlm.nih.gov/nuccore/328671415?report=fasta) | [MLOC_11773.1](http://apex.ipk-gatersleben.de/apex/f?p=284:20:::NO::P20_GENE_NAME:MLOC_11773.1) | ([Mangelsen et al., 2011](#_ENREF_29)) | 43.76 | morex_contig_1561802 CAJW011561802 |  |  |  |
| 5H | *BRASSINOSTEROID C-23 HYDROXYLASE (HvCPD)* | [KF360233.1](http://www.ncbi.nlm.nih.gov/nuccore/552355952) | [MLOC_10658.1](http://apex.ipk-gatersleben.de/apex/f?p=284:20:::NO::P20_GENE_NAME:MLOC_10658.1) | ([Dockter et al., 2014](#_ENREF_12)) | 44.02 | morex_contig_1559549 CAJW011559549 | (ppd-H1, SCRI_RS_188359  11_20697  SCRI_RS_139961  SCRI_RS_135425  (TIP, Hrv); 44.2- 44.24) |  | (ppd-H1, SCRI_RS_156396; 44.72) |
| 5H | *NARROW LEAF AND DWARF 1/* *TERMINAL FLOWER1 (HvND1/TFL1)* | [AK242601.1](http://www.ncbi.nlm.nih.gov/nuccore/AK242601) | [MLOC_17477.1](http://apex.ipk-gatersleben.de/apex/f?p=284:20:::NO::P20_GENE_NAME:MLOC_17477.1) | <http://rice.plantbiology.msu.edu/cgi-bin/ORF_infopage.cgi?orf=LOC_Os12g36890.1> | 44.09 | morex_contig_1576831 CAJW011576831 | (ppd-H1, SCRI_RS_188359  11_20697  SCRI_RS_139961  SCRI_RS_135425  (TIP, Hrv); 44.2- 44.24) |  | (ppd-H1, SCRI_RS_156396; 44.72) |
| 5H | *ASPARAGINE SYNTHASE1 (HvAS1)* | [AF307145.1](http://www.ncbi.nlm.nih.gov/nuccore/13925885) | [MLOC_63089.10](http://apex.ipk-gatersleben.de/apex/f?p=284:20:::NO::P20_GENE_NAME:MLOC_63089.10) | ([Møller et al., 2003](#_ENREF_32)) | 46.45 | morex_contig_47260 CAJW010047260 |  | (2R, SCRI_RS_162688 (AE); 46.32) |  |
| 5H | *DWARF KYUSHU 3 (HvD53)* | [AK240842](http://getentry.ddbj.nig.ac.jp/getentry/ddbj/AK240842?filetype=html) | [AK372211](http://apex.ipk-gatersleben.de/apex/f?p=284:20:::NO::P20_GENE_NAME:AK372211) | <http://rice.plantbiology.msu.edu/cgi-bin/ORF_infopage.cgi?orf=LOC_Os11g01330.1>  <http://rapdb.dna.affrc.go.jp/viewer/gene_detail/irgsp1?name=Os11t0104300-01;feature_id=2814952> | 46.59 | morex_contig_244827 CAJW010244827 |  | (2R, SCRI_RS_162688 (AE); 46.32) |  |
| 5H | *BRITTLE CULM12/* *GIBBERELLIN-DEFICIENT DWARF 1 (HvBC12/GGD1)* | [AK100974](http://www.ncbi.nlm.nih.gov/nuccore/AK100974) | [AK373790](http://apex.ipk-gatersleben.de/apex/f?p=284:20:::NO::P20_GENE_NAME:AK373790) | <http://rice.plantbiology.msu.edu/cgi-bin/ORF_infopage.cgi?orf=LOC_Os09g02650.1> | 47.22 | morex_contig_45441 CAJW010045441 | (ppd-H1,  11_11221  SCRI_RS_182435 (AP, Hrv); 47.57-47.70) | (2R, SCRI_RS_133042  SCRI_RS_13964  12_31183,  12_30538,  SCRI_RS_220389 (AE); 47.15-47.52) |  |
| 5H | *DWARF RICE WITH OVEREXPRESSION OF GIBBERELLIN-INDUCED GENE (HvDOG)* | [AK101969](http://getentry.ddbj.nig.ac.jp/getentry/ddbj/AK101969?filetype=html) | [AK359310](http://apex.ipk-gatersleben.de/apex/f?p=284:20:::NO::P20_GENE_NAME:AK359310) | <http://rice.plantbiology.msu.edu/cgi-bin/ORF_infopage.cgi?orf=LOC_Os08g39450.1> | 80.80 | morex_contig_1575121 CAJW011575121 | (ppd-H1,  11_20494  (Hrv); 80.81) |  |  |
| 5H | *MANY-NODED DWARF 6 (HvMND6)* | [HG965231](http://www.ncbi.nlm.nih.gov/nuccore/HG965231.1) | [MLOC_64838.2](http://apex.ipk-gatersleben.de/apex/f?p=284:20:::NO::P20_GENE_NAME:MLOC_64838.2) | ([Mascher et al., 2014](#_ENREF_30)) | 96.60 | morex_contig_49382 CAJW010049382 |  |  |  |
| 5H | *REGULATOR OF AXILLARY MERISTEMS3 (HvRAX3)* | [NP_190538.1](http://www.ncbi.nlm.nih.gov/protein/15229192) |  | ([Lin et al., 1999](#_ENREF_28)) | 130.69 | morex_contig_37892 | (ppd-H1,  SCRI_RS_148120  11_11024 (Hrv); 129.40) |  |  |
| 5H | *GIBBERELLIN 20 OXIDASE 1 (HvGA20ox1)* | [AY551428.1](http://www.ncbi.nlm.nih.gov/nuccore/AY551428.1) | [MLOC_16059.1](http://apex.ipk-gatersleben.de/apex/f?p=284:20:::NO::P20_GENE_NAME:MLOC_16059.1) | ([Jia et al., 2009](#_ENREF_20)) | 168.88 | morex_contig_1572260 CAJW011572260 |  |  |  |
| 6H | *HvCMF3* | [JQ791216](http://webblast.ipk-gatersleben.de/barley/blastresult.php?jobid=140125913497&opt=none) |  | ([Cockram et al., 2012](#_ENREF_7)) | 49.22 | morex_contig_56141 CAJW010056141 | (ppd-H1,  SCRI_RS_120783  12_30510  SCRI_RS_196459  SCRI_RS_182367  SCRI_RS_228181  SCRI_RS_182004 (Hrv); 49.08-49.22) | (2R, SCRI_RS_239889  SCRI_RS_238639  12_30510  SCRI_RS_228181  11_10013,  12_30316,  SCRI_RS_128460  SCRI_RS_196458  12_31274,  SCRI_RS_169829  SCRI_RS_187497  SCRI_RS_189254, (TIP), 49-49.22) |  |
| 6H | *HvCO7* | [AY082963](http://webblast.ipk-gatersleben.de/barley/blastresult.php?jobid=140125885789&opt=none) | [MLOC_38289.3](http://apex.ipk-gatersleben.de/apex/f?p=284:20:::NO::P20_GENE_NAME:MLOC_38289.3) | ([Griffiths et al., 2003](#_ENREF_14)) | 52.62 | morex_contig_2550116 CAJW012550116 | (ppd-H1,  SCRI_RS_166120  SCRI_RS_187832  SCRI_RS_131647 (Hrv); 52.5-52.9) | (2R, SCRI_RS_135049  SCRI_RS_131341 (AP), 52.83-  53.61) |  |
| 6H | *CYTOCHROME P450 (HvCYP734A7)* | [EU957215.1](http://www.ncbi.nlm.nih.gov/nuccore/EU957215.1) | [MLOC_72308.1](http://apex.ipk-gatersleben.de/apex/f?p=284:20:::NO::P20_GENE_NAME:MLOC_72308.1) | ([Alexandrov et al., 2009](#_ENREF_1)) | 54.81 | morex_contig_5616 CAJW010005616 | (ppd-H1,  12_30596  SCRI_RS_153502  SCRI_RS_174583  SCRI_RS_224695  SCRI_RS_122124  SCRI_RS_16796  SCRI_RS_142727  (Hrv); 54.85-54.90) |  |  |
| 6H | *HvCO5* | [AY082958](http://webblast.ipk-gatersleben.de/barley/blastresult.php?jobid=140125935217&opt=none) | [AK368809](http://apex.ipk-gatersleben.de/apex/f?p=284:20:::NO::P20_GENE_NAME:AK368809) | ([Griffiths et al., 2003](#_ENREF_14)) | 55.02 | morex_contig_243021 CAJW010243021 | (ppd-H1,  12_30120  SCRI_RS_168096  SCRI_RS_237750 (Hrv); 55.03) |  |  |
| 6H | *HvCry1a* | [DQ201149](http://webblast.ipk-gatersleben.de/barley/blastresult.php?jobid=140126090274&opt=none) | [MLOC_77423.4](http://apex.ipk-gatersleben.de/apex/f?p=284:20:::NO::P20_GENE_NAME:MLOC_77423.4) | ([Szucs et al., 2006](#_ENREF_42)) | 55.02 | morex_contig_75574 CAJW010075574 | (ppd-H1,  SCRI_RS_153502  SCRI_RS_174583  SCRI_RS_224695  SCRI_RS_122124  SCRI_RS_16796  SCRI_RS_142727  12_30120  SCRI_RS_168096  SCRI_RS_237750 (Hrv); 54.89-55.03) |  |  |
| 6H | *HvCry2* | [DQ201155](http://webblast.ipk-gatersleben.de/barley/blastresult.php?jobid=140126139716&opt=none#BL_ORD_ID:2883973) | [AK363503](http://apex.ipk-gatersleben.de/apex/f?p=284:20:::NO::P20_GENE_NAME:AK363503) | ([Szucs et al., 2006](#_ENREF_42)) | 55.02 | morex_contig_141897 CAJW010141897 | (ppd-H1,  SCRI_RS_153502  SCRI_RS_174583  SCRI_RS_224695  SCRI_RS_122124  SCRI_RS_16796  SCRI_RS_142727  12_30120  SCRI_RS_168096  SCRI_RS_237750 (Hrv); 54.89-55.03) |  |  |
| 6H | *HvPRR1/HvTOC1* | [JQ791234](http://webblast.ipk-gatersleben.de/barley/blastresult.php?jobid=140126006288&opt=none) | [MLOC_52387.1](http://apex.ipk-gatersleben.de/apex/f?p=284:20:::NO::P20_GENE_NAME:MLOC_52387.1) | ([Cockram et al., 2012](#_ENREF_7)) | 55.38 | morex_contig_37494 CAJW010037494 | (ppd-H1,  SCRI_RS_138529  (Hrv); 55.38) | (2R, SCRI_RS_146992 (AP), 55.84) |  |
| 6H | *HvCry1b* | [DQ201152](http://webblast.ipk-gatersleben.de/barley/blastresult.php?jobid=140126123570&opt=none) | [MLOC_64083.1](http://apex.ipk-gatersleben.de/apex/f?p=284:20:::NO::P20_GENE_NAME:MLOC_64083.1) | ([Szucs et al., 2006](#_ENREF_42)) | 59.06 | morex_contig_48345 CAJW010048345 |  | (2R, SCRI_RS_146992 (AP), 55.84) |  |
| 6H | *HvCO14* | [JQ791244](http://webblast.ipk-gatersleben.de/barley/blastresult.php?jobid=140126037462&opt=none) | [MLOC_51238.1](http://apex.ipk-gatersleben.de/apex/f?p=284:20:::NO::P20_GENE_NAME:MLOC_51238.1) | ([Cockram et al., 2012](#_ENREF_7)) | 67.91 | morex_contig_367999 CAJW010367999 | (ppd-H1,  SCRI_RS_136724 (Hrv); 67.92) |  |  |
| 6H | *HvCO2* | [AF490469](http://webblast.ipk-gatersleben.de/barley/blastresult.php?jobid=140126056975&opt=none) | [MLOC_75496.6](http://apex.ipk-gatersleben.de/apex/f?p=284:20:::NO::P20_GENE_NAME:MLOC_75496.6) | ([Griffiths et al., 2003](#_ENREF_14)) | 68.20 | morex_contig_6805 CAJW010006805 |  | (2R, SCRI_RS_204363 (HD), 68.9) |  |
| 6H | *HvCO11* | [JQ791238](http://webblast.ipk-gatersleben.de/barley/blastresult.php?jobid=140126074279&opt=none) | [AK368809](http://apex.ipk-gatersleben.de/apex/f?p=284:20:::NO::P20_GENE_NAME:AK368809) | ([Cockram et al., 2012](#_ENREF_7)) | 69.26 | morex_contig_1577721 CAJW011577721 |  | (2R, SCRI_RS_204363 (HD), 68.9) |  |
| 6H | *CONSTITUTIVELY PHOTOMORPHOGENIC 1 (HvCOP1)* | [BAK06015.1](http://www.ncbi.nlm.nih.gov/protein/326499049?report=genbank&log$=protalign&blast_rank=69&RID=908J11WB01R) |  | ([Matsumoto et al., 2011](#_ENREF_31)) | 88.6 | morex_contig_7610 | (ppd-H1,  11_11294 (Hrv); 88.88) |  |  |
| 6H | *TREHALOSE-6-PHOSPHATE SYNTHASE 2 (HvTPS2)* | [HM446021.1](http://www.ncbi.nlm.nih.gov/nuccore/HM446021.1) | [AK366091](http://apex.ipk-gatersleben.de/apex/f?p=284:20:::NO::P20_GENE_NAME:AK366091) | ([Mangelsen et al., 2011](#_ENREF_29)) | 95.04 | morex_contig_1581754 CAJW011581754 | (Ppd-H1, ppd-H1  SCRI_RS_224910 SCRI_RS_159133  SCRI_RS_173142  11_10015 ( Prod, non-p) 94.9 95) |  |  |
| 7H | *ENT-KAURENOIC ACID HYDROXYLASE 1 (HvKAO1)* | [AF326277.1](http://www.ncbi.nlm.nih.gov/nuccore/AF318500) | [MLOC_54352.1](http://apex.ipk-gatersleben.de/apex/f?p=284:20:::NO::P20_GENE_NAME:MLOC_54352.1) | ([Helliwell et al., 2001](#_ENREF_16)) | 1.71 | morex_contig_39067 CAJW010039067 |  |  |  |
| 7H | *DWARF 3 (HvD3), CYTOCHROME P450 88A3* | [AK069429](http://www.ncbi.nlm.nih.gov/nuccore/AK069429) | [MLOC_54352.1](http://apex.ipk-gatersleben.de/apex/f?p=284:20:::NO::P20_GENE_NAME:MLOC_54352.1) | ([Consortium et al., 2003](#_ENREF_9)) | 1.91 | morex_contig_39067 CAJW010039067 |  |  |  |
| 7H | *DWARF AND LOW-TILLERING (HvDLT)* | [AK106449.1](http://www.ncbi.nlm.nih.gov/nuccore/AK106449) | [MLOC_31327.1](http://apex.ipk-gatersleben.de/apex/f?p=284:20:::NO::P20_GENE_NAME:MLOC_31327.1) | <http://rice.plantbiology.msu.edu/cgi-bin/ORF_infopage.cgi?orf=LOC_Os06g03710.1> | 12.75 | morex_contig_2179585 |  |  |  |
| 7H | *MORE AXILLARY BRANCHES 2 (HvMAX2)* | [AK065478](http://getentry.ddbj.nig.ac.jp/getentry/ddbj/AK065478?filetype=html) | [MLOC_4044.5](http://apex.ipk-gatersleben.de/apex/f?p=284:20:::NO::P20_GENE_NAME:MLOC_4044.5) | <http://rice.plantbiology.msu.edu/cgi-bin/ORF_infopage.cgi?orf=LOC_Os06g06050.1> | 29.95 | morex_contig_134615 CAJW01013461 |  |  |  |
| 7H | *SOLUBLE STARCH SYNTHASE (HvSSI)* | [FN179374.1](http://www.ncbi.nlm.nih.gov/nuccore/229610850?report=fasta) | [MLOC_70501.1](http://apex.ipk-gatersleben.de/apex/f?p=284:20:::NO::P20_GENE_NAME:MLOC_70501.1) | ([Radchuk et al., 2009](#_ENREF_34)) | 37.60 | morex_contig_58077 CAJW010058077 |  |  |  |
| 7H | *SUCROSE SYNTHASE(HvSS)* | [X65871.1](http://www.ncbi.nlm.nih.gov/nuccore/19105?report=fasta) | [MLOC_11770.1](http://apex.ipk-gatersleben.de/apex/f?p=284:20:::NO::P20_GENE_NAME:MLOC_11770.1) | ([de la Hoz et al., 1992](#_ENREF_11)) | 54.39 | morex_contig_1561797 CAJW011561797 | (Ppd-H1 (Prod) SCRI_RS_230478, 54.8 ) |  |  |
| 7H | *WEALTHY FARMERS PANICLE 1/*  *IDEAL PLANT ARCHITECTURE 1/SQUAMOSA PROMOTER BINDING PROTEIN-LIKE 14 (HvWFP1/HvIPA1/HvSPL14)* | [GU136674.1](http://www.ncbi.nlm.nih.gov/nuccore/GU136674.1) | [AK363115](http://apex.ipk-gatersleben.de/apex/f?p=284:20:::NO::P20_GENE_NAME:AK363115) | ([Jiao et al., 2010](#_ENREF_21)) | 70.50 | morex_contig_45350 CAJW010045350 | (ppd-H1,  11_20314 (Hrv); 70.3) | (2R, SCRI_RS_152635 (AE) ; 70.57) | (6R, 12_10581; 71.2) |
| 7H | *DWARF 35 (HvD35), CYTOCHROME P450 701A6* | [AK066285](http://getentry.ddbj.nig.ac.jp/getentry/ddbj/AK066285?filetype=html) | [AK369327](http://apex.ipk-gatersleben.de/apex/f?p=284:20:::NO::P20_GENE_NAME:AK369327) | ([Itoh et al., 2004](#_ENREF_18)) | 77.40 | morex_contig_1575857 CAJW011575857 | (ppd-H1,  SCRI_RS_207238 (Hrv); 77.3) |  |  |
| 7H | *MONOCULM 1 (MOC1)* | [AY242058.1](http://www.ncbi.nlm.nih.gov/nuccore/AY242058.1) | [AK364492](http://apex.ipk-gatersleben.de/apex/f?p=284:20:::NO::P20_GENE_NAME:AK364492) | ([Li et al., 2003](#_ENREF_27)) | 77.53 | morex_contig_7090 |  |  |  |
| 7H | *REGULATOR OF AXILLARY MERISTEMS2 (HvRAX2)* | [NP_181226.1](http://www.ncbi.nlm.nih.gov/protein/15228049) |  | ([Lin et al., 1999](#_ENREF_28);[Müller et al., 2006](#_ENREF_33)) | 89.13 | morex_contig_274295 CAJW010274295 |  | (2R, SCRI_RS_14491,  SCRI_RS_222286 (AE); 91.92) |  |
| 7H | *ENHANCER OF SHOOT REGENERATION (HvESR1)* | [CV063802.1](http://www.ncbi.nlm.nih.gov/nucest/CV063802.1) | [MLOC_10878.1](http://apex.ipk-gatersleben.de/apex/f?p=284:20:::NO::P20_GENE_NAME:MLOC_10878.1) | ([Ali et al., 2000](#_ENREF_2)) | 120.39 | morex_contig_39720 CAJW010039720 |  |  |  |
| 7H | *BRASSINOSTEROID DEFICIENT DWARF 2/ DIMINUTO, DWARF1 (HvBRD2/HvDIM/HvDWF1)* | [AK111949](http://getentry.ddbj.nig.ac.jp/getentry/ddbj/AK111949?filetype=html) | [MLOC_52405.2](http://apex.ipk-gatersleben.de/apex/f?p=284:20:::NO::P20_GENE_NAME:MLOC_52405.2) | <http://rice.plantbiology.msu.edu/cgi-bin/ORF_infopage.cgi?orf=LOC_Os10g25780.1> | 140.65 | morex_contig_37512 CAJW010037512 | (ppd-H1,  SCRI_RS_169268 (Hrv); 140.7) | (6R, 11_10174 (Hrv); 140.9) |  |
| 7H | *MORE AXILLARY BRANCHES 1 (HvMAX1)* | [JX566699.1](http://www.ncbi.nlm.nih.gov/nuccore/JX566699.1) | [MLOC_69509.1](http://apex.ipk-gatersleben.de/apex/f?p=284:20:::NO::P20_GENE_NAME:MLOC_69509.1) | ([Challis et al., 2013](#_ENREF_4)) , | 77.41 (unpublished data) | morex_contig_55549 CAJW010055549 |  |  |  |
| 7H | *DWARF27 (HvD27)* | [FJ641055](http://www.ncbi.nlm.nih.gov/nucleotide/514809529?report=genbank&log$=nuclalign&blast_rank=2&RID=97YPA8KT01R) | [MLOC_67450.8](http://apex.ipk-gatersleben.de/apex/f?p=284:20:::NO::P20_GENE_NAME:MLOC_67450.8) | <http://blast.ncbi.nlm.nih.gov/Blast.cgi#alnHdr_514809529> | 89.52 | morex_contig_53102 |  |  |  |

Two-rowed (2R); six-rowed (6R); photoperiod-sensitive (*Ppd-H1*) and reduced photoperiodic sensitivity (*ppd-H1*). Awn primordium (AP); tipping (TIP); heading (HD); anther extrusion (AE) and harvest (Hrv). SCRI_RS_21483: marker name; 55.5: marker position (cM) anchored by physical map positions based on Barke x Morex RILs POPSEQ population (Mascher et al. 2013)

Alexandrov, N., Brover, V., Freidin, S., Troukhan, M., Tatarinova, T., Zhang, H., Swaller, T., Lu, Y.-P., Bouck, J., Flavell, R., and Feldmann, K. (2009). Insights into corn genes derived from large-scale cDNA sequencing. *Plant Molecular Biology* 69**,** 179-194. 10.1007/s11103-008-9415-4:

Ali, S., Holloway, B., and Taylor, W. (2000). Normalisation of cereal endosperm EST libraries for structural and functional genomic analysis. *Plant Molecular Biology Reporter* 18**,** 123-132. 10.1007/BF02824020:

Booker, J., Auldridge, M., Wills, S., Mccarty, D., Klee, H., and Leyser, O. (2004). MAX3/CCD7 Is a Carotenoid Cleavage Dioxygenase Required for the Synthesis of a Novel Plant Signaling Molecule. *Current Biology* 14**,** 1232-1238. <http://dx.doi.org/10.1016/j.cub.2004.06.061:>

Challis, R.J., Hepworth, J., Mouchel, C., Waites, R., and Leyser, O. (2013). A Role for MORE AXILLARY GROWTH1 (MAX1) in Evolutionary Diversity in Strigolactone Signaling Upstream of MAX2. *Plant Physiology* 161**,** 1885-1902. 10.1104/pp.112.211383:

Chandler, P.M., Marion-Poll, A., Ellis, M., and Gubler, F. (2002). Mutants at the Slender1 Locus of Barley cv Himalaya. Molecular and Physiological Characterization. *Plant Physiology* 129**,** 181-190. 10.1104/pp.010917:

Cho, J.I., Ryoo, N., Ko, S., Lee, S.K., Lee, J., Jung, K.H., Lee, Y.H., Bhoo, S.H., Winderickx, J., An, G., Hahn, T.R., and Jeon, J.S. (2006). Structure, expression, and functional analysis of the hexokinase gene family in rice (*Oryza sativa* L.). *Planta* 224**,** 598-611. 10.1007/s00425-006-0251-y:

Cockram, J., Thiel, T., Steuernagel, B., Stein, N., Taudien, S., Bailey, P.C., and O'sullivan, D.M. (2012). Genome dynamics explain the evolution of flowering time CCT domain gene families in the Poaceae. *PLoS One* 7**,** e45307. 10.1371/journal.pone.0045307:

Comadran, J., Kilian, B., Russell, J., Ramsay, L., Stein, N., Ganal, M., Shaw, P., Bayer, M., Thomas, W., Marshall, D., Hedley, P., Tondelli, A., Pecchioni, N., Francia, E., Korzun, V., Walther, A., and Waugh, R. (2012). Natural variation in a homolog of *Antirrhinum CENTRORADIALIS* contributed to spring growth habit and environmental adaptation in cultivated barley. *Nat Genet* 44**,** 1388-1392. 10.1038/ng.2447:

Consortium, T.R.F.-L.C., Team:, N.I.O.a.S.R.F.-L.C.P., Kikuchi, S., Satoh, K., Nagata, T., Kawagashira, N., Doi, K., Kishimoto, N., Yazaki, J., Ishikawa, M., Yamada, H., Ooka, H., Hotta, I., Kojima, K., Namiki, T., Ohneda, E., Yahagi, W., Suzuki, K., Li, C.J., Ohtsuki, K., Shishiki, T., Sequencing, F.O.a.O.I.S.G., Group:, A., Otomo, Y., Murakami, K., Iida, Y., Sugano, S., Fujimura, T., Suzuki, Y., Tsunoda, Y., Kurosaki, T., Kodama, T., Masuda, H., Kobayashi, M., Xie, Q., Lu, M., Narikawa, R., Sugiyama, A., Mizuno, K., Yokomizo, S., Niikura, J., Ikeda, R., Ishibiki, J., Kawamata, M., Yoshimura, A., Miura, J., Kusumegi, T., Oka, M., Ryu, R., Ueda, M., Matsubara, K., Riken:, Kawai, J., Carninci, P., Adachi, J., Aizawa, K., Arakawa, T., Fukuda, S., Hara, A., Hashidume, W., Hayatsu, N., Imotani, K., Ishii, Y., Itoh, M., Kagawa, I., Kondo, S., Konno, H., Miyazaki, A., Osato, N., Ota, Y., Saito, R., Sasaki, D., Sato, K., Shibata, K., Shinagawa, A., Shiraki, T., Yoshino, M., and Hayashizaki, Y. (2003). Collection, Mapping, and Annotation of Over 28,000 cDNA Clones from japonica Rice. *Science* 301**,** 376-379. 10.1126/science.1081288:

Dabbert, T., Okagaki, R.J., Cho, S., Heinen, S., Boddu, J., and Muehlbauer, G.J. (2010). The genetics of barley low-tillering mutants: *low number of tillers-1* (*lnt1*). *Theor Appl Genet* 121**,** 705-715. 10.1007/s00122-010-1342-5:

De La Hoz, P.S., Vicente-Carbajosa, J., Mena, M., and Carbonero, P. (1992). Homologous sucrose synthase genes in barley (Hordeum vulgare) are located in chromosomes 7H (syn. 1 and 2H Evidence for a gene translocation? *FEBS Letters* 310**,** 46-50. <http://dx.doi.org/10.1016/0014-5793(92)81143-A:>

Dockter, C., Gruszka, D., Braumann, I., Druka, A., Druka, I., Franckowiak, J., Gough, S.P., Janeczko, A., Kurowska, M., Lundqvist, J., Lundqvist, U., Marzec, M., Matyszczak, I., Muller, A.H., Oklestkova, J., Schulz, B., Zakhrabekova, S., and Hansson, M. (2014). Induced variations in brassinosteroid genes define barley height and sturdiness, and expand the green revolution genetic toolkit. *Plant Physiol* 166**,** 1912-1927. 10.1104/pp.114.250738:

Faure, S., Higgins, J., Turner, A., and Laurie, D.A. (2007). The *FLOWERING LOCUS T*-like gene family in barley (*Hordeum vulgare*). *Genetics* 176**,** 599-609. 10.1534/genetics.106.069500:

Griffiths, S., Dunford, R.P., Coupland, G., and Laurie, D.A. (2003). The evolution of CONSTANS-like gene families in barley, rice, and Arabidopsis. *Plant Physiol* 131**,** 1855-1867. 10.1104/pp.102.016188:

Guan, J.C., Koch, K.E., Suzuki, M., Wu, S., Latshaw, S., Petruff, T., Goulet, C., Klee, H.J., and Mccarty, D.R. (2012). Diverse Roles of Strigolactone Signaling in Maize Architecture and the Uncoupling of a Branching-Specific Subnetwork. *Plant Physiology* 160**,** 1303-1317. 10.1104/pp.112.204503:

Helliwell, C.A., Chandler, P.M., Poole, A., Dennis, E.S., and Peacock, W.J. (2001). The CYP88A cytochrome P450, ent-kaurenoic acid oxidase, catalyzes three steps of the gibberellin biosynthesis pathway. *Proceedings of the National Academy of Sciences* 98**,** 2065-2070. 10.1073/pnas.98.4.2065:

Houston, K., Mckim, S.M., Comadran, J., Bonar, N., Druka, I., Uzrek, N., Cirillo, E., Guzy-Wrobelska, J., Collins, N.C., Halpin, C., Hansson, M., Dockter, C., Druka, A., and Waugh, R. (2013). Variation in the interaction between alleles of *HvAPETALA2* and microRNA172 determines the density of grains on the barley inflorescence. *Proc Natl Acad Sci U S A* 110**,** 16675-16680. 10.1073/pnas.1311681110:

Itoh, H., Tatsumi, T., Sakamoto, T., Otomo, K., Toyomasu, T., Kitano, H., Ashikari, M., Ichihara, S., and Matsuoka, M. (2004). A Rice Semi-Dwarf Gene, Tan-Ginbozu (D35), Encodes the Gibberellin Biosynthesis Enzyme, ent-Kaurene Oxidase. *Plant Molecular Biology* 54**,** 533-547. 10.1023/B:PLAN.0000038261.21060.47:

Itoh, H., Ueguchi-Tanaka, M., Sentoku, N., Kitano, H., Matsuoka, M., and Kobayashi, M. (2001). Cloning and functional analysis of two gibberellin 3β-hydroxylase genes that are differently expressed during the growth of rice. *Proceedings of the National Academy of Sciences* 98**,** 8909-8914. 10.1073/pnas.141239398:

Jia, Q., Zhang, J., Westcott, S., Zhang, X.-Q., Bellgard, M., Lance, R., and Li, C. (2009). GA-20 oxidase as a candidate for the semidwarf gene sdw1/denso in barley. *Functional & Integrative Genomics* 9**,** 255-262. 10.1007/s10142-009-0120-4:

Jiao, Y., Wang, Y., Xue, D., Wang, J., Yan, M., Liu, G., Dong, G., Zeng, D., Lu, Z., Zhu, X., Qian, Q., and Li, J. (2010). Regulation of *OsSPL14* by *OsmiR156* defines ideal plant architecture in rice. *Nat Genet* 42**,** 541-544. 10.1038/ng.591:

Kebrom, T.H., Chandler, P.M., Swain, S.M., King, R.W., Richards, R.A., and Spielmeyer, W. (2012). Inhibition of tiller bud outgrowth in the tin mutant of wheat is associated with precocious internode development. *Plant Physiol* 160**,** 308-318. 10.1104/pp.112.197954:

Kebrom, T.H., Spielmeyer, W., and Finnegan, E.J. (2013). Grasses provide new insights into regulation of shoot branching. *Trends Plant Sci* 18**,** 41-48. 10.1016/j.tplants.2012.07.001:

Komatsuda, T., Pourkheirandish, M., He, C., Azhaguvel, P., Kanamori, H., Perovic, D., Stein, N., Graner, A., Wicker, T., Tagiri, A., Lundqvist, U., Fujimura, T., Matsuoka, M., Matsumoto, T., and Yano, M. (2007). Six-rowed barley originated from a mutation in a homeodomain-leucine zipper I-class homeobox gene. *Proc Natl Acad Sci U S A* 104**,** 1424-1429. 10.1073/pnas.0608580104:

Koppolu, R., Anwar, N., Sakuma, S., Tagiri, A., Lundqvist, U., Pourkheirandish, M., Rutten, T., Seiler, C., Himmelbach, A., Ariyadasa, R., Youssef, H.M., Stein, N., Sreenivasulu, N., Komatsuda, T., and Schnurbusch, T. (2013). *Six-rowed spike4* (*Vrs4*) controls spikelet determinacy and row-type in barley. *Proc Natl Acad Sci U S A* 110**,** 13198-13203. 10.1073/pnas.1221950110:

Kyozuka, J., Konishi, S., Nemoto, K., Izawa, T., and Shimamoto, K. (1998). Down-regulation of *RFL*, the *FLO*/*LFY* homolog of rice, accompanied with panicle branch initiation. *Proc Natl Acad Sci U S A* 95**,** 1979-1982.

Li, X., Qian, Q., Fu, Z., Wang, Y., Xiong, G., Zeng, D., Wang, X., Liu, X., Teng, S., Hiroshi, F., Yuan, M., Luo, D., Han, B., and Li, J. (2003). Control of tillering in rice. *Nature* 422**,** 618-621. <http://www.nature.com/nature/journal/v422/n6932/suppinfo/nature01518_S1.html:>

Lin, X., Kaul, S., Rounsley, S., Shea, T.P., Benito, M.-I., Town, C.D., Fujii, C.Y., Mason, T., Bowman, C.L., Barnstead, M., Feldblyum, T.V., Buell, C.R., Ketchum, K.A., Lee, J., Ronning, C.M., Koo, H.L., Moffat, K.S., Cronin, L.A., Shen, M., Pai, G., Van Aken, S., Umayam, L., Tallon, L.J., Gill, J.E., Adams, M.D., Carrera, A.J., Creasy, T.H., Goodman, H.M., Somerville, C.R., Copenhaver, G.P., Preuss, D., Nierman, W.C., White, O., Eisen, J.A., Salzberg, S.L., Fraser, C.M., and Venter, J.C. (1999). Sequence and analysis of chromosome 2 of the plant Arabidopsis thaliana. *Nature* 402**,** 761-768.

Mangelsen, E., Kilian, J., Harter, K., Jansson, C., Wanke, D., and Sundberg, E. (2011). Transcriptome Analysis of High-Temperature Stress in Developing Barley Caryopses: Early Stress Responses and Effects on Storage Compound Biosynthesis. *Molecular Plant* 4**,** 97-115. <http://dx.doi.org/10.1093/mp/ssq058:>

Mascher, M., Jost, M., Kuon, J.E., Himmelbach, A., Assfalg, A., Beier, S., Scholz, U., Graner, A., and Stein, N. (2014). Mapping-by-sequencing accelerates forward genetics in barley. *Genome Biol* 15**,** R78. 10.1186/gb-2014-15-6-r78:

Matsumoto, T., Tanaka, T., Sakai, H., Amano, N., Kanamori, H., Kurita, K., Kikuta, A., Kamiya, K., Yamamoto, M., Ikawa, H., Fujii, N., Hori, K., Itoh, T., and Sato, K. (2011). Comprehensive Sequence Analysis of 24,783 Barley Full-Length cDNAs Derived from 12 Clone Libraries. *Plant Physiology* 156**,** 20-28. 10.1104/pp.110.171579:

Møller, M.G., Taylor, C., Rasmussen, S.K., and Holm, P.B. (2003). Molecular cloning and characterisation of two genes encoding asparagine synthetase in barley (Hordeum vulgare L.). *Biochimica et Biophysica Acta (BBA) - Gene Structure and Expression* 1628**,** 123-132. <http://dx.doi.org/10.1016/S0167-4781(03)00137-4:>

Müller, D., Schmitz, G., and Theres, K. (2006). Blind Homologous R2R3 Myb Genes Control the Pattern of Lateral Meristem Initiation in Arabidopsis. *The Plant Cell Online* 18**,** 586-597. 10.1105/tpc.105.038745:

Radchuk, V.V., Borisjuk, L., Sreenivasulu, N., Merx, K., Mock, H.-P., Rolletschek, H., Wobus, U., and Weschke, W. (2009). Spatiotemporal Profiling of Starch Biosynthesis and Degradation in the Developing Barley Grain. *Plant Physiology* 150**,** 190-204. 10.1104/pp.108.133520:

Saisho, D., Tanno, K., Chono, M., Honda, I., Kitano, H., and Takeda, K. (2004). Spontaneous Brassinolide-insensitive barley mutants 'uzu' adapted to East Asia. *Breeding Science* 54**,** 409-416. 10.1270/jsbbs.54.409:

Sakamoto, T., Miura, K., Itoh, H., Tatsumi, T., Ueguchi-Tanaka, M., Ishiyama, K., Kobayashi, M., Agrawal, G.K., Takeda, S., Abe, K., Miyao, A., Hirochika, H., Kitano, H., Ashikari, M., and Matsuoka, M. (2004). An Overview of Gibberellin Metabolism Enzyme Genes and Their Related Mutants in Rice. *Plant Physiology* 134**,** 1642-1653. 10.1104/pp.103.033696:

Sakuma, S., Pourkheirandish, M., Matsumoto, T., Koba, T., and Komatsuda, T. (2010). Duplication of a well-conserved homeodomain-leucine zipper transcription factor gene in barley generates a copy with more specific functions. *Functional & Integrative Genomics* 10**,** 123-133. 10.1007/s10142-009-0134-y:

Sivitz, A.B., Reinders, A., and Ward, J.M. (2005). Analysis of the Transport Activity of Barley Sucrose Transporter HvSUT1. *Plant and Cell Physiology* 46**,** 1666-1673. 10.1093/pcp/pci182:

Soderlund, C., Descour, A., Kudrna, D., Bomhoff, M., Boyd, L., Currie, J., Angelova, A., Collura, K., Wissotski, M., Ashley, E., Morrow, D., Fernandes, J., Walbot, V., and Yu, Y. (2009). Sequencing, Mapping, and Analysis of 27,455 Maize Full-Length cDNAs. *PLoS Genet* 5**,** e1000740. 10.1371/journal.pgen.1000740:

Spielmeyer, W., Ellis, M., Robertson, M., Ali, S., Lenton, J., and Chandler, P. (2004). Isolation of gibberellin metabolic pathway genes from barley and comparative mapping in barley, wheat and rice. *Theoretical and Applied Genetics* 109**,** 847-855. 10.1007/s00122-004-1689-6:

Sun, C., Palmqvist, S., Olsson, H., Borén, M., Ahlandsberg, S., and Jansson, C. (2003). A Novel WRKY Transcription Factor, SUSIBA2, Participates in Sugar Signaling in Barley by Binding to the Sugar-Responsive Elements of the iso1 Promoter. *The Plant Cell* 15**,** 2076-2092. 10.1105/tpc.014597:

Szucs, P., Karsai, I., Von Zitzewitz, J., Meszaros, K., Cooper, L.L., Gu, Y.Q., Chen, T.H., Hayes, P.M., and Skinner, J.S. (2006). Positional relationships between photoperiod response QTL and photoreceptor and vernalization genes in barley. *Theor Appl Genet* 112**,** 1277-1285. 10.1007/s00122-006-0229-y:

Tanabe, S., Ashikari, M., Fujioka, S., Takatsuto, S., Yoshida, S., Yano, M., Yoshimura, A., Kitano, H., Matsuoka, M., Fujisawa, Y., Kato, H., and Iwasaki, Y. (2005). A Novel Cytochrome P450 Is Implicated in Brassinosteroid Biosynthesis via the Characterization of a Rice Dwarf Mutant, dwarf11, with Reduced Seed Length. *The Plant Cell Online* 17**,** 776-790. 10.1105/tpc.104.024950:

Tavakol, E., Okagaki, R., Verderio, G., Shariati, J.V., Hussien, A., Bilgic, H., Scanlon, M.J., Todt, N.R., Close, T.J., Druka, A., Waugh, R., Steuernagel, B., Ariyadasa, R., Himmelbach, A., Stein, N., Muehlbauer, G.J., and Rossini, L. (2015). The barley *Uniculme4* gene encodes a BLADE-ON-PETIOLE-like protein that controls tillering and leaf patterning. *Plant Physiol* 168**,** 164-174. 10.1104/pp.114.252882:

Weschke, W., Panitz, R., Gubatz, S., Wang, Q., Radchuk, R., Weber, H., and Wobus, U. (2003). The role of invertases and hexose transporters in controlling sugar ratios in maternal and filial tissues of barley caryopses during early development. *The Plant Journal* 33**,** 395-411. 10.1046/j.1365-313X.2003.01633.x:

Weschke, W., Panitz, R., Sauer, N., Wang, Q., Neubohn, B., Weber, H., and Wobus, U. (2000). Sucrose transport into barley seeds: molecular characterization of two transporters and implications for seed development and starch accumulation. *The Plant Journal* 21**,** 455-467. 10.1046/j.1365-313x.2000.00695.x:

Yang, Y.H., Zhang, F.M., and Ge, S. (2009). Evolutionary rate patterns of the Gibberellin pathway genes. *BMC Evol Biol* 9**,** 206. 10.1186/1471-2148-9-206:

Youssef, H.M., Koppolu, R., and Schnurbusch, T. (2012). Re-sequencing of vrs1 and int-c loci shows that labile barleys (Hordeum vulgare convar. labile) have a six-rowed genetic background. *Genetic Resources and Crop Evolution* 59**,** 1319-1328. 10.1007/s10722-011-9759-5:

Zhang, H., Sreenivasulu, N., Weschke, W., Stein, N., Rudd, S., Radchuk, V., Potokina, E., Scholz, U., Schweizer, P., Zierold, U., Langridge, P., Varshney, R.K., Wobus, U., and Graner, A. (2004). Large-scale analysis of the barley transcriptome based on expressed sequence tags. *The Plant Journal* 40**,** 276-290. 10.1111/j.1365-313X.2004.02209.x:
