# Supplementary material for: The Genetic Architecture of Barley Plant Stature
Source: Front Genet. 2016 Jun 24;7:117. doi: 10.3389/fgene.2016.00117 (PMC4919324; doi:10.3389/fgene.2016.00117)
Supplement: Supplementary file 5 [file Image1.pdf]

The genetic architecture of barley plant stature
Frontiers in Genetics 7
DOI: 10.3389/fgene.2016.00117

Ahmad M. Alqudah[1]✉; Ravi Koppolu[1]; Gizaw M. Wolde[1]; Andreas Graner[2]; Thorsten Schnurbusch[1]✉

[1]HEISENBERG-Research Group Plant Architecture,
[2]Research Group Genome Diversity,
Leibniz Institute of Plant Genetics and Crop Plant Research (IPK),
Corrensstrasse 3, OT Gatersleben, D-06466 Stadt Seeland, Germany

✉Corresponding authors:
Ahmad M. Alqudah,
Tel: +49-39482-5826, email: alqudah@ipk-gatersleben.de
PD Dr. Thorsten Schnurbusch,
Tel: +49-39482-5341, Fax: +49-39482-5595, email: thor@ipk-gatersleben.de
HEISENBERG-Research Group Plant Architecture
Leibniz Institute of Plant Genetics and Crop Plant Research (IPK)
Corrensstrasse 3, OT Gatersleben, D-06466 Stadt Seeland, Germany

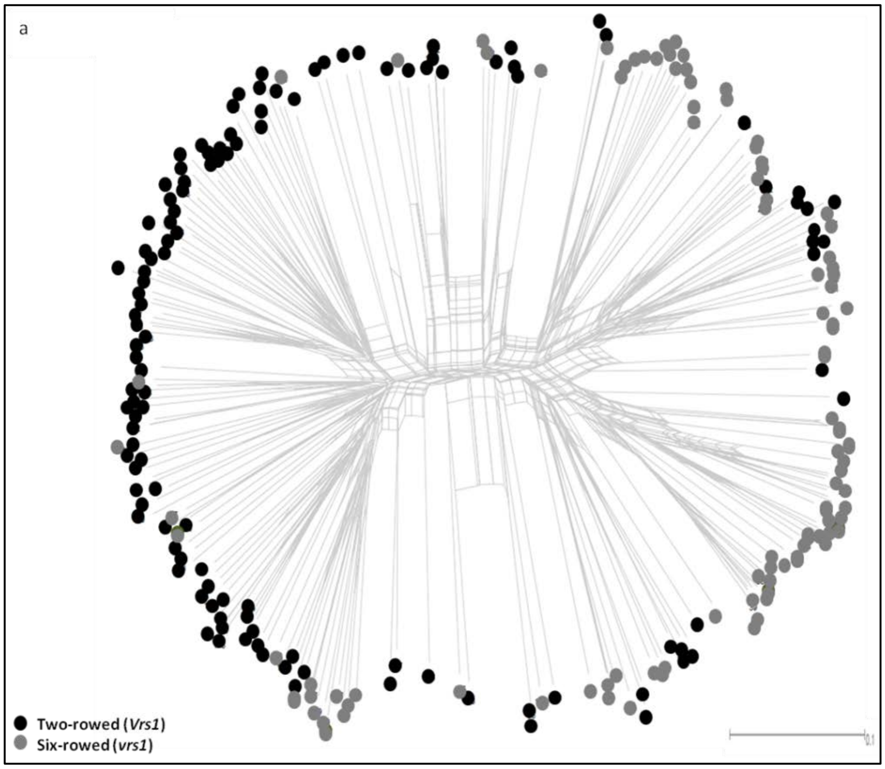

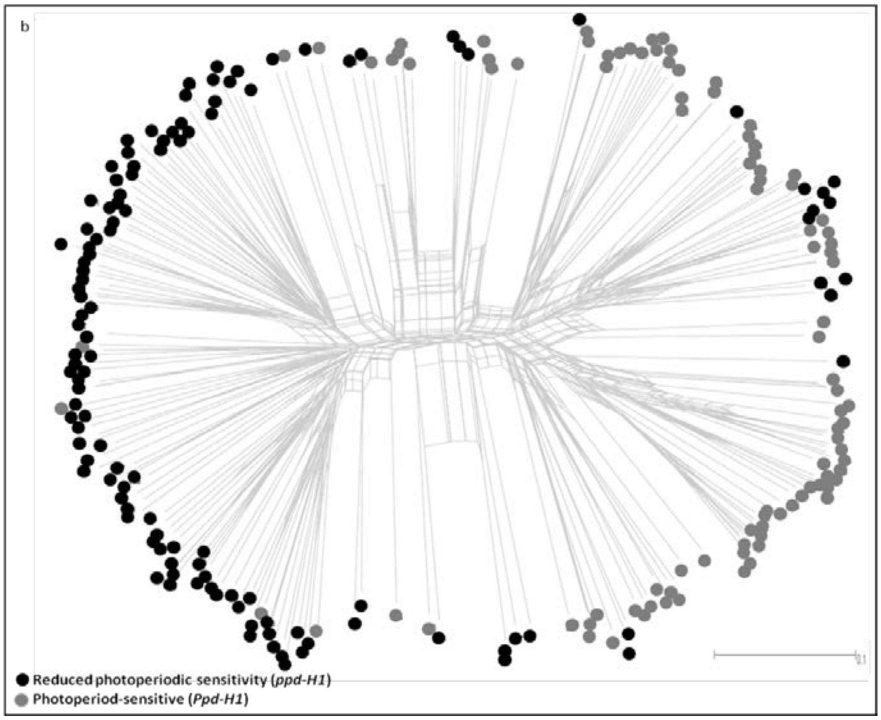

Figure S1: Population structure of 218 spring barley accessions based on 6355 SNPs information. (a.) 125 accessions are phenotypically classified as two- and 93 accessions as six-rowed. (b.) 95 accessions show photoperiod response (*Ppd-H1*) to long day conditions and 123 accessions show reduced photoperiod response (*ppd-H1*).
