# Supplementary material for: The Genetic Architecture of Barley Plant Stature
Source: Front Genet. 2016 Jun 24;7:117. doi: 10.3389/fgene.2016.00117 (PMC4919324; doi:10.3389/fgene.2016.00117)
Supplement: Supplementary file 6 [file Image2.pdf]

The genetic architecture of barley plant stature

Frontiers in Genetics 7
DOI: 10.3389/fgene.2016.00117

Ahmad M. Alqudah[1]✉; Ravi Koppolu[1]; Gizaw M. Wolde[1]; Andreas Graner[2]; Thorsten Schnurbusch[1]✉

[1]HEISENBERG-Research Group Plant Architecture,
[2]Research Group Genome Diversity,
Leibniz Institute of Plant Genetics and Crop Plant Research (IPK),
Corrensstrasse 3, OT Gatersleben, D-06466 Stadt Seeland, Germany

✉Corresponding authors:
Ahmad M. Alqudah,
Tel: +49-39482-5826, email: alqudah@ipk-gatersleben.de
PD Dr. Thorsten Schnurbusch,
Tel: +49-39482-5341, Fax: +49-39482-5595, email: thor@ipk-gatersleben.de
HEISENBERG-Research Group Plant Architecture
Leibniz Institute of Plant Genetics and Crop Plant Research (IPK)
Corrensstrasse 3, OT Gatersleben, D-06466 Stadt Seeland, Germany

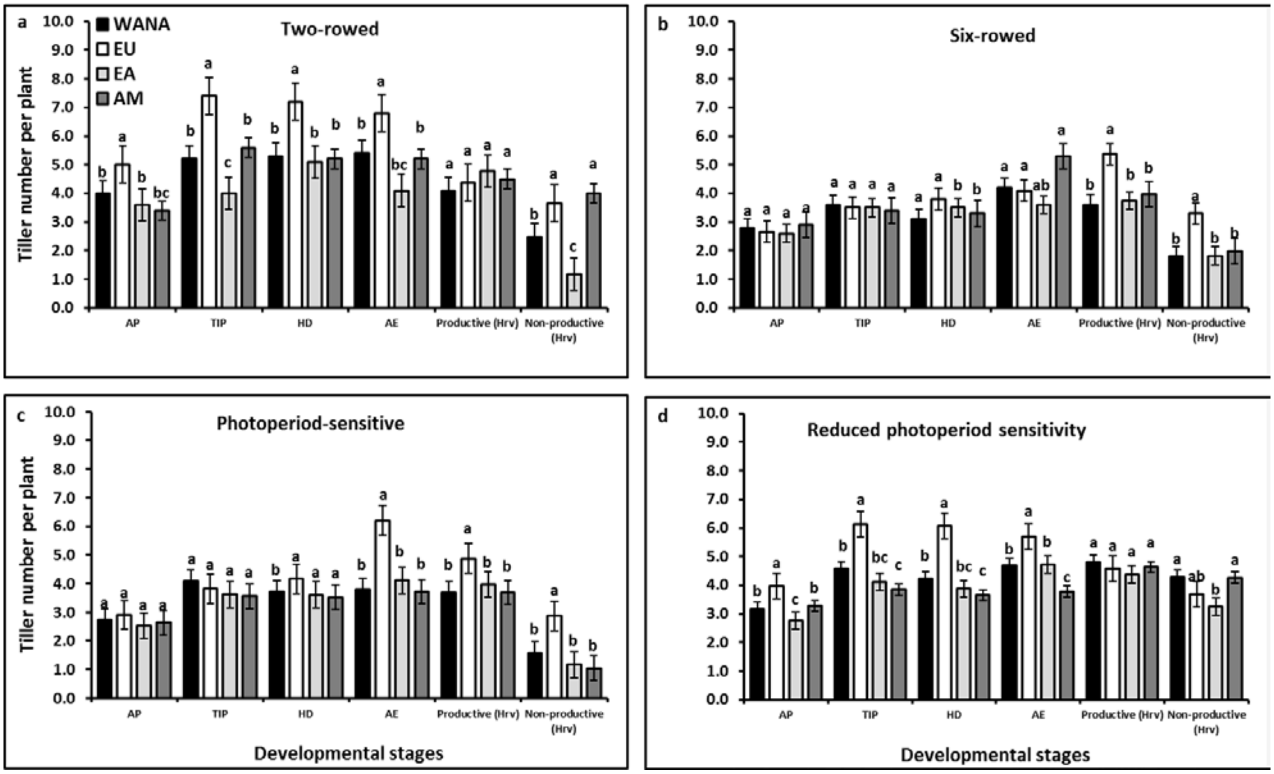

Figure S2: Total tiller number per plant based on origins of accessions in two-rowed (a), six-rowed (b), photoperiod-sensitive (c) and reduced photoperiod sensitivity (d). The same letters for each developmental stage are not significantly different at $P \leq 0.05$ according to LSD. Bars indicate LSD. Three biological replicates were used from each accession at each pre-anthesis developmental stage and six biological replicates were used for counting productive and non-productive tiller at harvest stage. (n = 125 and 93 for two- and six-rowed barleys, respectively; and n= 95 and 123 for photoperiod sensitive and reduced photoperiod sensitivity barley, respectively).

AP: awn primordium (Alqudah and Schnurbusch, 2014); TIP: tipping, Z49; HD: heading, Z55; AE: anther extrusion, Z65; Hrv: Harvesting (Zadoks et al., 1974).
Developmental stages calculated based on thermal time $^{o}C\times D^{-1}$ (GDD).

Alqudah, A.M., and Schnurbusch, T. (2014). Awn primordium to tipping is the most decisive developmental phase for spikelet survival in barley. *Funct Plant Biol* 41**,** 424-436. 10.1071/FP13248:

Zadoks, J.C., Chang, T.T., and Konzak, C.F. (1974). A decimal code for the growth stages of cereals. *Weed Research* 14**,** 415-421.
