# Supplementary material for: The Genetic Architecture of Barley Plant Stature
Source: Front Genet. 2016 Jun 24;7:117. doi: 10.3389/fgene.2016.00117 (PMC4919324; doi:10.3389/fgene.2016.00117)
Supplement: Supplementary file 9 [file Image5.pdf]

The genetic architecture of barley plant stature
Frontiers in Genetics 7
DOI: 10.3389/fgene.2016.00117

Ahmad M. Alqudah[1✉]; Ravi Koppolu[1]; Gizaw M. Wolde[1]; Andreas Graner[2]; Thorsten Schnurbusch[1✉]

[1]HEISENBERG-Research Group Plant Architecture,
[2]Research Group Genome Diversity,
Leibniz Institute of Plant Genetics and Crop Plant Research (IPK),
Corrensstrasse 3, OT Gatersleben, D-06466 Stadt Seeland, Germany

✉Corresponding authors:
Ahmad M. Alqudah,
Tel: +49-39482-5826, email: alqudah@ipk-gatersleben.de
PD Dr. Thorsten Schnurbusch,
Tel: +49-39482-5341, Fax: +49-39482-5595, email: thor@ipk-gatersleben.de
HEISENBERG-Research Group Plant Architecture
Leibniz Institute of Plant Genetics and Crop Plant Research (IPK)
Corrensstrasse 3, OT Gatersleben, D-06466 Stadt Seeland, Germany

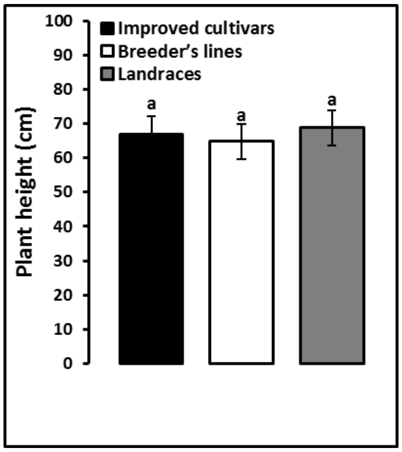

Figure S5: Plant height (cm) at harvest based on biological status. The same letters are not significantly different at $P \leq 0.05$ according to LSD. Bars indicate LSD. Three biological replicates were used from each genotype at each developmental stage. (n = 149, 57 and 18 for cultivars, landraces and barleys breeding lines, respectively).
