# Supplementary material for: The Genetic Architecture of Barley Plant Stature
Source: Front Genet. 2016 Jun 24;7:117. doi: 10.3389/fgene.2016.00117 (PMC4919324; doi:10.3389/fgene.2016.00117)
Supplement: Supplementary file 10 [file Image6.pdf]

The genetic architecture of barley plant stature
Frontiers in Genetics 7
DOI: 10.3389/fgene.2016.00117

Ahmad M. Alqudah[1✉]; Ravi Koppolu[1]; Gizaw M. Wolde[1]; Andreas Graner[2]; Thorsten Schnurbusch[1✉]

[1]HEISENBERG-Research Group Plant Architecture,
[2]Research Group Genome Diversity,
Leibniz Institute of Plant Genetics and Crop Plant Research (IPK),
Corrensstrasse 3, OT Gatersleben, D-06466 Stadt Seeland, Germany

✉Corresponding authors:
Ahmad M. Alqudah,
Tel: +49-39482-5826, email: alqudah@ipk-gatersleben.de
PD Dr. Thorsten Schnurbusch,
Tel: +49-39482-5341, Fax: +49-39482-5595, email: thor@ipk-gatersleben.de
HEISENBERG-Research Group Plant Architecture
Leibniz Institute of Plant Genetics and Crop Plant Research (IPK)
Corrensstrasse 3, OT Gatersleben, D-06466 Stadt Seeland, Germany

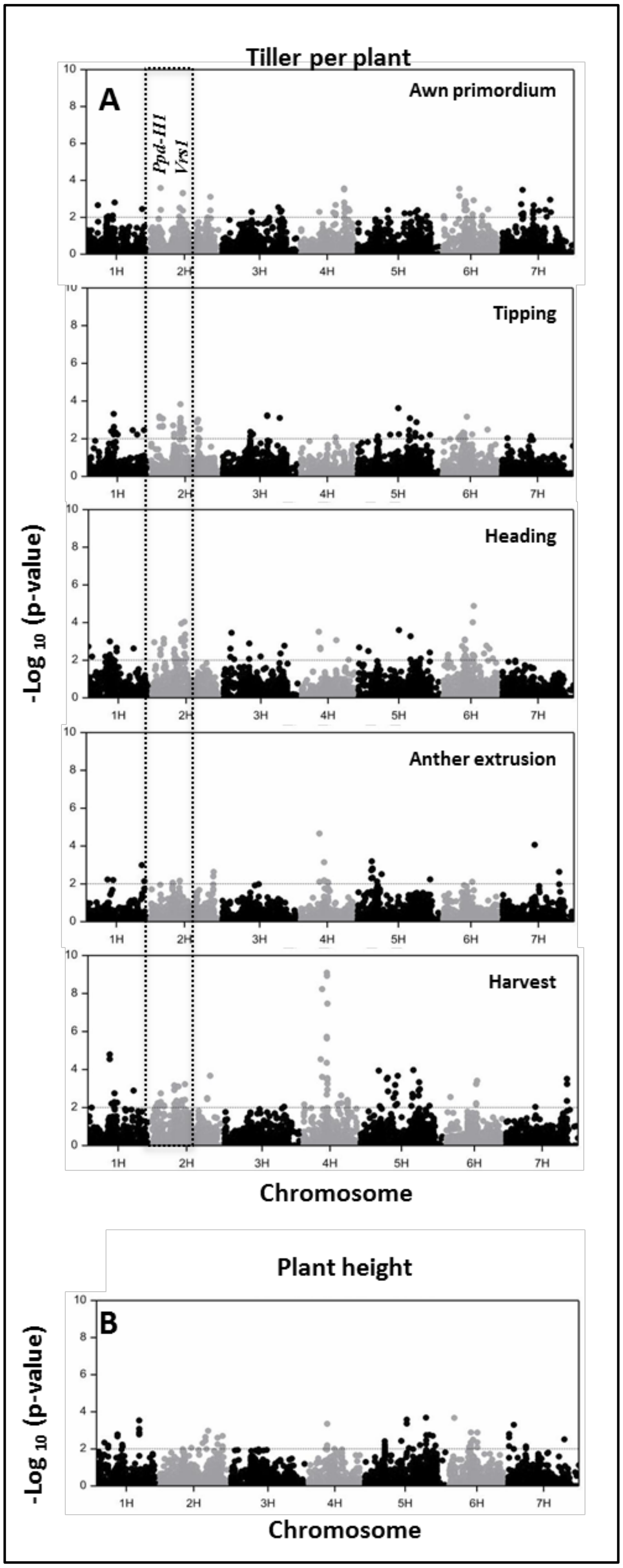

Figure S6: Manhattan plots of associations signals. The figures summarize GWAS results obtained for A) total tiller number per plant and B) plant height in whole spring barley collection using the iSelect 9K SNP platform. *n* = 218 accessions.
